# The Proteome Profiles of the Cerebellum of Juvenile, Adult and Aged Rats—An Ontogenetic Study

**DOI:** 10.3390/ijms160921454

**Published:** 2015-09-07

**Authors:** Michael Wille, Antje Schümann, Andreas Wree, Michael Kreutzer, Michael O. Glocker, Grit Mutzbauer, Oliver Schmitt

**Affiliations:** 1 Department of Anatomy, Gertrudenstr. 9, 18055 Rostock, Germany; E-Mails: michael.wille@uni-rostock.de (M.W.); antje.schuemann@uni-rostock.de (A.S.); andreas.wree@med.uni-rostock.de (A.W.); 2 Proteome Center Rostock, Schillingallee 69, 18055 Rostock, Germany; E-Mails: michael.kreutzer@med.uni-rostock.de (M.K.); michael.glocker@med.uni-rostock.de (M.O.G.); 3 Department of Pathology, Josef-Schneider-Str. 2, 97080 Würzburg, Germany; E-Mail: grit.lessner@uni-wuerzburg.de

**Keywords:** proteomics, rat, brain, cerebellum, development

## Abstract

In this study, we searched for proteins that change their expression in the cerebellum (Ce) of rats during ontogenesis. This study focuses on the question of whether specific proteins exist which are differentially expressed with regard to postnatal stages of development. A better characterization of the microenvironment and its development may result from these study findings. A differential two-dimensional polyacrylamide gel electrophoresis (2DE) and matrix-assisted laser desorption/ionization time-of-flight mass spectrometry (MALDI-TOF-MS) analysis of the samples revealed that the number of proteins of the functional classes differed depending on the developmental stages. Especially members of the functional classes of biosynthesis, regulatory proteins, chaperones and structural proteins show the highest differential expression within the analyzed stages of development. Therefore, members of these functional protein groups seem to be involved in the development and differentiation of the Ce within the analyzed development stages. In this study, changes in the expression of proteins in the Ce at different postnatal developmental stages (postnatal days (P) 7, 90, and 637) could be observed. At the same time, an identification of proteins which are involved in cell migration and differentiation was possible. Especially proteins involved in processes of the biosynthesis and regulation, the dynamic organization of the cytoskeleton as well as chaperones showed a high amount of differentially expressed proteins between the analyzed dates.

## 1. Introduction

The aim of this study was to analyze the differential proteome of the rat Ce at different developmental stages (7-day-old juvenile rats (P7), 90-day-old adult rats (P90) and 637-day-old aged rats (P637)).

The Ce is located in the hindbrain region and stems from the dorsolateral part of the alar plate. It develops from the rostral limbs at the level of the rhombencephalon. It is responsible for balance and posture as well as the coordination of gait, voluntary movement and is important in control of gaze [1]. Regarding the ontogenesis of the Ce, additional information about the detailed development of this brain region, including changes of the brains mass, neuronal development and morphological changes is listed in the supplements (see Figure S1, accompanying text).

As mentioned above, the Ce is essential for different tasks in fine coordination and other important motor functions. Also in Parkinson’s disease, a chronic progressive neurodegenerative disorder characterized by resting tremor, slowness of movements, rigidity, gait disturbance and postural instability [2], involvement of Ce in producing some motoric symptoms can be observed [3,4]. For example, different studies identified reciprocal connections between the basal ganglia and the Ce. Also functional and morphological changes in the Ce were detected related to akinesia, rigidity, tremor, gait disturbance, dyskinesia and some non-motor symptoms. Besides, pathological changes in the Ce might be induced by dopaminergic degeneration [2]. These findings provide a basis to explain the role of the Ce in Parkinson’s disease. Based on this, an experimental investigation for optimized therapeutic strategies, e.g., stem cell transplantation in experimental models of Parkinson’s disease, is still essential [5]. Previous studies [6,7] showed a different development of transplanted progenitor cells in neural and glial cell types of the neonatal and adult striata. A better characterization of the development of the microenvironment at the proteome level, including the determination of factors which are important for the development of this region, may support a better understanding of survival and differentiation of transplanted progenitor cells.

The lysosomal storage disease Niemann-Pick disease, type C1 (NPC1) is caused by a mutation of the NPC1 gene and results in a neurological disorder that is specifically related to the cerebellum. This neurodegenerative disorder leads to a huge loss of Purkinje neurons for which there is no definitive therapy yet. Main differences in the expression of proteins related to this disorder were found in the category of proteins involved in glucose metabolism, detoxification/oxidative stress and Alzheimer disease-related proteins. Furthermore, members of the fatty acid binding protein family, including FABP3, FABP5 and FABP7, show an altered expression in the NPC1 cerebellum [8]. Further aspects of the developing Ce are different changes of expression in the granular cell cytoskeleton. The neuronal cytoskeleton is composed of three main elements: micro- and neurofilaments as well as microtubules [9,10,11]. For the microfilaments, these polymers consist of actin with associated actin-regulatory proteins [12,13]. Neurofilaments consist of three polypeptides (NFH, NFM, NFL) and microtubules are polymers formed from dimers of α- and β-tubulin with associated proteins (microtubule-associated proteins (MAPS)). These proteins play a major role in the transport of new components down axons and dendrites and are important in cell division, cell migration and the formation of the complex morphology of neurons in the developing brain [14,15]. Hence, proteins associated with neurofilaments are involved in granule cell development and appearance of the characteristic morphology of mature granule cells. They must be involved in complex regulation of the composition and interactions between the components of the granule cell cytoskeleton [16].

In addition, expression and activity changes of glycolytic enzymes, proteins integrated in their biogenesis and metabolic components of the energy metabolism present another main aspect during growth and development of the rat brain. As an example, the activity of phospho-fructokinase (a key enzyme which catalyzes the conversion of fructose-6-phosphate to fructose-1,6-bisphosphate and which also represents the rate-limiting step in the glycolysis) is detectable in fetal brains at the twelfth day of gestation. It shows little change in enzyme activity in the brain from 5 days before birth to 8 days after birth. During this period, activity was 30%–40% of the normal value found in an adult brain. After 12 days, a rapid increase in activity to 21 days occurred [17]. Furthermore, the activities of additional glycolytic enzymes (hexokinase [18], aldolase [19], lactate-dehydrogenase [20], for example) do show substantial increases during maturation of the rat brain. As Leong and Clark stated [21], because of the correlation between the developmental sequences of glycolytic enzymes with the neurophylogenetic development of the brain, this adds support to the hypothesis that the development of the potential for glycolysis in the brain is a necessary prerequisite for the development of neurological competence.

In this study, proteins from the Ce of juvenile (P7) adult (P90) and aged rats (P637) were separated by 2DE and identified by MALDI-TOF-MS. An identification of differential protein expression changes during ontogenesis will be presented. As yet, protein expressions in the developing proteome are not fully understood and have not been published elsewhere.

## 2. Results

An identification of the differential protein expression between the groups of different ages (P7, P90 and P637) was accomplished by using 2DE with subsequent gel-matching, spot-warping and differential spot analysis combined with protein identification by MALDI-TOF-MS.

**Figure 1:**
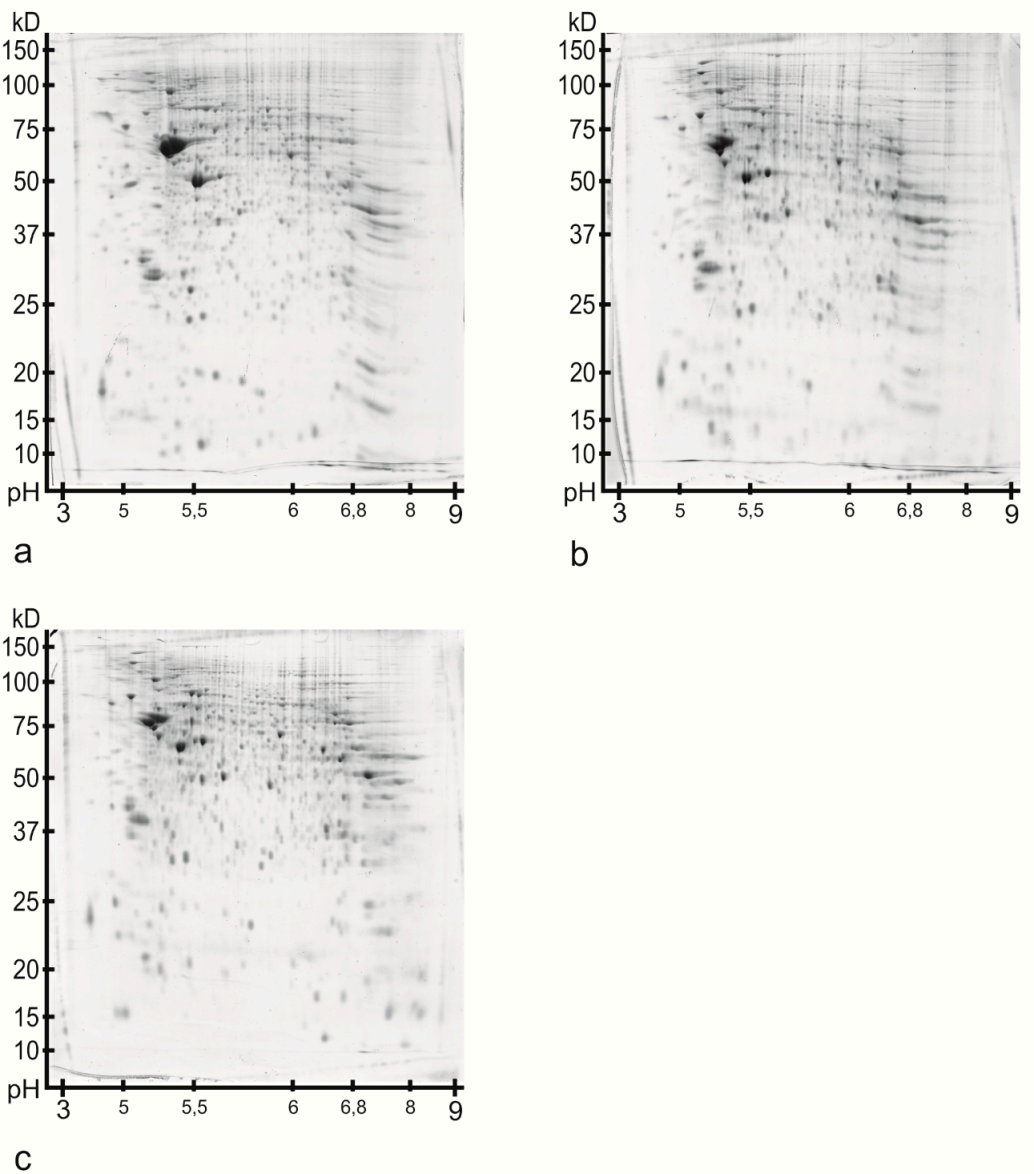
Overview of the reference gel images from the Ce. The Coomassie blue stainings of the gels have similar intensities. The 2DE-gel image of a P7 animal is shown in (**a**); In (**b**) the 2DE-gel image of a P90 animal is presented; (**c**) Shows the 2DE-gel image of an old P637 rat.

Figure 1 presents the images of the reference gels for the different development stages ((a) P7, (b) P90 and (c) P637). The spot compositions turn out to be comparable. An amount of 901 (±73) spots was detected in the six gels of P7, 637 (±108) spots in the six gels of P90 and 775 (±145) spots in the six gels of P637.

**Figure 2:**
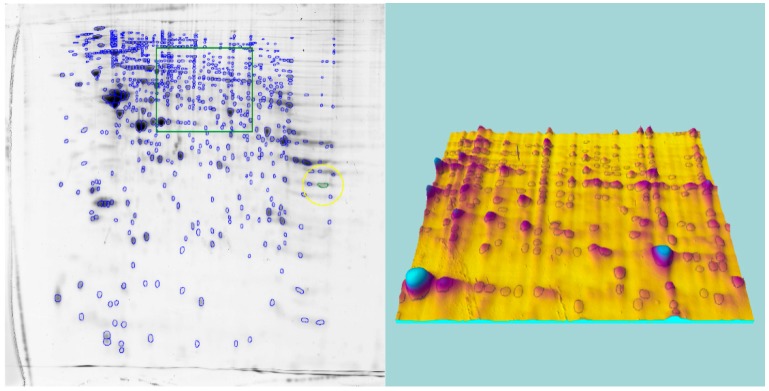
In order to perform an accurate segmentation of spots, the manual editing of spots was augmented by a 3D-visualization. This procedure has been applied to all reference gels as well as to all template gels. An example of the latter is presented here. The green box on the left indicates a region which is visualized as a 3D-view on the right side. The yellow circle is a temporary marker of the PG200.

To obtain an impression of the quality of our manual segmentation of each spot in the Ce gels, a segmented gel image in combination with the 3D-visualization tool of Progenesis PG200 is presented (Figure 2).

The proteins which showed a differential expression were divided into 13 functional groups. In the following, a description of the groups of proteins is given which showed a higher amount of differential expression than the others. The description of the remaining analyzed proteins of the other functional groups is listed in the supplements. The differentially expressed proteins were divided into 13 functional groups. In the following, a description of the groups of proteins is given which showed a higher amount of differential expression than the others. The description of the remaining analyzed proteins of the other functional groups is listed in the supplements.

**Figure 3:**
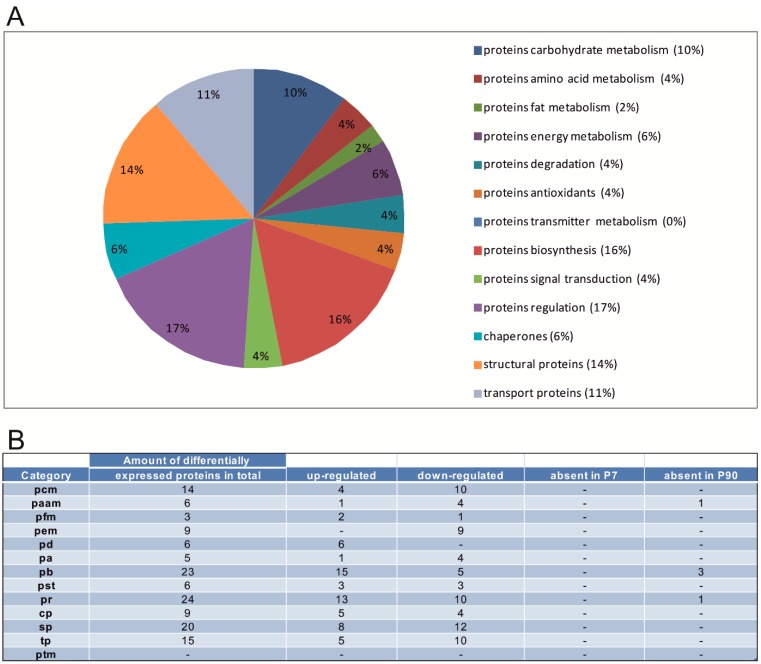
Differential expression of proteins in the Ce at P7. (**A**) Relative frequencies of proteins in the Ce that are differentially expressed (P7 *vs.* P90); (**B**) Number of differentially expressed proteins of different protein categories within the Ce (P7) *vs.* (P90). (Abbreviations for Figure 3B and Figure 4B, pcm: proteins carbohydrate metabolism, paam: proteins amino acid metabolism, pfm: proteins fat metabolism, pem: proteins energy metabolism, pd: proteins degradation, pa: proteins antioxidants, ptm: proteins transmitter metabolism, pb: proteins biosynthesis, pst: proteins signal transduction, pr: proteins regulation, cp: chaperones, sp: structural proteins, tp: transport proteins).

**Figure 4:**
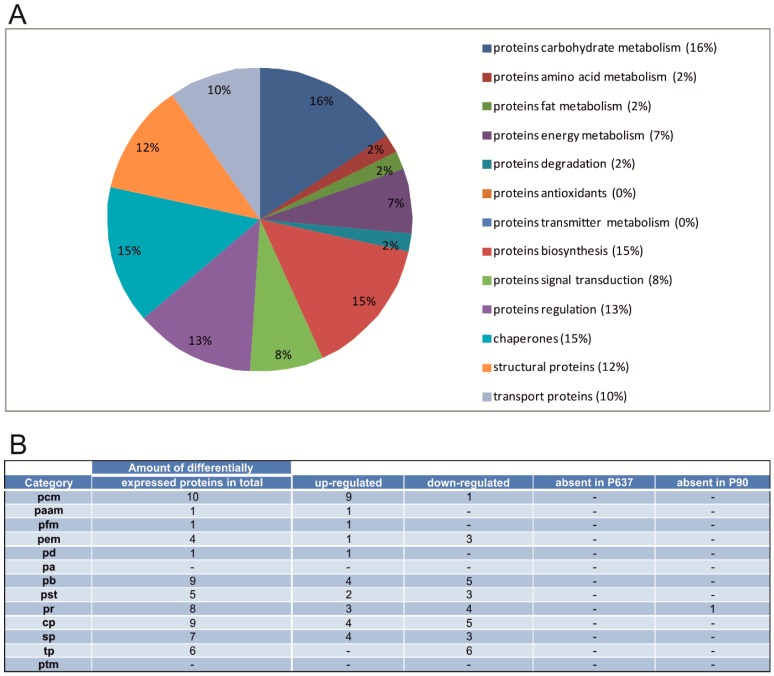
Differential expression of proteins in the Ce at P637. (**A**) Relative frequencies of proteins in the Ce that are differentially expressed (P637 *vs.* P90); (**B**) Number of differentially expressed proteins of different protein categories within the Ce (P637) *vs.* (P90). Same abbreviations performed as in Figure 3B.

In the following, a detailed presentation of the up- and down-regulation of single proteins of categories which showed the most differential expression changes, setting P90 as base-line expression (see Figure 3B and Figure 4B) is given. Based on the masspectrometic analysis, in the following, proteins which show an unambiguous differential expression are described in detail (see Table 1). The results of the multi condition coverage method as well as the Mann-Whitney *U*-test are presented. Proteins which were detected in different spots or which expression change is not distinct are marked with an (*) within the text. All analyzed proteins, identified by peptide mass fingerprinting, are arranged by their function and presented in Table S1.

**Table 1:** Proteins identified by peptide mass fingerprinting in spots from Coomassie-stained gels arranged by their function. Accession: Accession number from UniProtKB/Swiss-Prot; Entry name: Entry name from UniProtKB/Swiss-Prot; Gene name: from UniProtKB/Swiss-Prot; Regulation (P7)/Regulation (P637): Differential expression in comparison to P90; Control/PD: Spot volume quotient; H: Number of differential expressed spots per biological replicate; Score: Mascot MOWSE-score; Qm: Number of mass values (tryptic peptides) assigned to the identified protein; MW: Theoretical molecular mass (Da); pI: Theoretical pI value taken from the Mascot report. The full description for the cellular location is listed in the list of abbreviations.

Accession	Entry Name	Gene Name	Functional Group/Protein Names	Cell Localization	Regulation (P7)		Regulation (P637)		Control/PD	H	Score	Qm	*M*_W_	pI
					(SVQ ≤ 0.6)	*p*-Value	(SVQ ≥ 1.67)	*p*-Value	(P90/P7, P637)				(Calc.)	
			**Carbohydrate Metabolism**											
P08461	ODP2_RAT	*Dlat*	Dihydrolipoyllysine-residue acetyltransferase component of pyruvate dehydrogenase complex, mitochondrial	Mmt	-	-	up	0.0062	0.32	5	241	28	67,637	8.76
P04764	ENOA_RAT	*Eno1*	Alpha-enolase	Cp, M	down	0.1745	-	-	1.90	5	107	12	47,440	6.16
P07323	ENOG_RAT	*Eno2*	Gamma-enolase	Cp, M	down	0.3367	-	-	2.22	6	272	32	47,510	5.03
P04797	G3P_RAT	*Gapdh*	Glyceraldehyde-3-phosphate dehydrogenase	Cp, Nc	down	0.0250	-	-	3.09	6	262	34	36,090	8.14
P04636	MDHM_RAT	*Mdh2*	Malate dehydrogenase, mitochondrial	Mmt	-	-	up	0.1441	0.49	5	242	24	36,117	8.93
Q5XI78	ODO1_RAT	*Ogdh*	2-oxoglutarate dehydrogenase, mitochondrial	Mmt	down	0.0190	-	-	1.93	4	158	18	117,419	6.3
P49432	ODPB_RAT	*Pdhb*	Pyruvate dehydrogenase E1 component subunit beta, mitochondrial	Mmt	down	0.0039	-	-	5.80	6	139	18	39,299	6.2
D3Z955	D3Z955_RAT	*Pgm2l1*	Phosphoglucomutase 2-like 1	Cts	up	0.0250	-	-	0.52	6	126	17	71,102	6.09
P50137	TKT_RAT	*Tkt*	Transketolase	Nc	down	0.0065	-	-	2.09	6	339	35	68,342	7.23
			**Amino Acid Metabolism**											
Q6Q0N1	CNDP2_RAT	*Cndp2*	Cytosolic non-specific dipeptidase	Cp	-	-	up	0.0106	0.48	5	205	29	53,116	5.43
Q4V7C6	GUAA_RAT	*Gmps*	GMP synthase (glutamine-hydrolyzing)	Cp	up	0.0062	-	-	0.52	5	130	17	77,507	6.21
P14882	PCCA_RAT	*Pcca*	Propionyl-CoA carboxylase alpha chain, mitochondrial	Mmt	down	0.0283	-	-	2.14	5	262	32	82,198	7.59
			**Fat Metabolism**											
Q5XI22	THIC_RAT	*Acat2*	Acetyl-CoA acetyltransferase, cytosolic	Cp	-	-	up	0.0209	0.42	4	180	16	41,538	6.86
O35263	PA1B3_RAT	*Pafah1b3*	Platelet-activating factor acetylhydrolase IB subunit gamma	Cp	up	0.0062	-	-	0.27	6	135	11	25,961	6.42
Q568Z9	PHYIP_RAT	*Phyhip*	Phytanoyl-CoA hydroxylase-interacting protein	Pers	up	0.0105	-	-	0.54	4	160	17	38,101	6.53
			**Energy Metabolism**											
P15999	ATPA_RAT	*Atp5a1*	ATP synthase subunit alpha, mitochondrial	Mim, M	down	0.0285	up	0.0001	3.49	5	295	38	59,831	9.22
P31399	ATP5H_RAT	*Atp5h*	ATP synthase subunit d, mitochondrial	Mito, Mm	down	0.0039	-	-	2.70	6	148	13	18,809	6.17
Q561S0	NDUAA_RAT	*Ndufa10*	NADH dehydrogenase (ubiquinone) 1 alpha subcomplex subunit 10, mitochondrial	Mmt	down	0.0190	-	-	3.09	4	261	21	40,753	7.64
Q641Y2	NDUS2_RAT	*Ndufs2*	NADH dehydrogenase [ubiquinone] iron-sulfur protein 2, mitochondrial	Mim	down	0.0163	-	-	2.46	6	247	31	52,927	6.52
D3ZG43	D3ZG43_RAT	*Ndufs3*	NADH dehydrogenase (Ubiquinone) Fe-S protein 3 (Predicted), isoform CRA_c	Mito, Mm	down	0.0039	-	-	3.29	6	280	21	20,190	6.32
P19234	NDUV2_RAT	*Ndufv2*	NADH dehydrogenase (ubiquinone) flavoprotein 2, mitochondrial	Mim	down	0.0472	-	-	2.48	5	138	13	27,703	6.23
P19234	NDUV2_RAT	*Ndufv2*	NADH dehydrogenase [ubiquinone] flavoprotein 2, mitochondrial	Mim	down	0.0472	-	-	2.48	5	138	13	27,703	6.23
			**Degratory Proteins**											
P60901	PSA6_RAT	*Psma6*	Proteasome subunit alpha type-6	Cp, Nc	up	0.0039	-	-	0.48	6	173	16	27,838	6.34
P40112	PSB3_RAT	*Psmb3*	Proteasome subunit beta type-3	Cp, Nc	up	0.0176	-	-	0.27	5	124	12	23,234	6.15
D4A640	D4A640_RAT	*Psmb4*	Proteasome subunit beta type	Cp, Nc	up	0.0065	-	-	0.56	6	102	7	25,858	5.97
Q9JHW0	PSB7_RAT	*Psmb7*	Proteasome subunit beta type-7	Cp, Nc	up	0.0039	-	-	0.44	6	120	10	30,250	8.13
Q4V8E2	Q4V8E2_RAT	*Psmd14*	Proteasome (Prosome, macropain) 26S subunit, non-ATPase, 14	P, Np	up	0.0881	-	-	0.56	4	64	7	34,726	6.06
			**Antioxidants**											
O08557	DDAH1_RAT	*Ddah1*	*N*(G),*N*(G)-dimethylarginine dimethylaminohydrolase 1	Mito, Cp	up	0.0176	-	-	0.44	5	283	26	31,805	5.75
Q9Z1B2	GSTM5_RAT	*Gstm5*	Glutathione *S*-transferase Mu 5	Cp	down	0.0065	-	-	2.92	6	232	22	27,067	6.33
Q9Z339	GSTO1_RAT	*Gsto1*	Glutathione *S*-transferase omega-1	Cp, Cts	down	0.0547	-	-	2.27	6	81	10	27,936	6.25
Q9Z0V6	PRDX3_RAT	*Prdx3*	Thioredoxin-dependent peroxide reductase, mitochondrial	Mito	down	0.0104	-	-	1.87	6	72	8	28,563	7.14
O35244	PRDX6_RAT	*Prdx6*	Peroxiredoxin-6	Cp, Lyso	down	0.0039	-	-	3.75	6	207	18	24,860	5.64
			**Biosynthesis**											
Q6P7P5	BZW1_RAT	*Bzw1*	Basic leucine zipper and W2 domain-containing protein 1	Cp	up	0.0550	-	-	0.52	4	77	10	48,184	5.75
Q4KM73	KCY_RAT	*Cmpk1*	UMP-CMP kinase	Nc, Cp	up	0.0163	-	-	0.55	6	149	10	22,383	5.66
Q6P7P5	BZW1_RAT	*Bzw1*	Basic leucine zipper and W2 domain-containing protein 1	Cp	up	0.0550	-	-	0.52	4	77	10	48,184	5.75
Q4KM73	KCY_RAT	*Cmpk1*	UMP-CMP kinase	Nc, Cp	up	0.0163	-	-	0.55	6	149	10	22,383	5.66
P62630	EF1A1_RAT	*Eef1a1*	Elongation factor 1-alpha 1	Cp, Nc	up	0.5839	-	-	0.44	5	73	10	50,424	9.1
Q68FR6	EF1G_RAT	*Eef1g*	Elongation factor 1-gamma	Rs, Cts	up	0.0550	-	-	0.37	6	235	27	50,371	6.31
Q5RKI1	IF4A2_RAT	*Eif4a2*	Eukaryotic initiation factor 4A-II	Cts	-	-	down	0.0090	5.16	5	196	28	46,601	5.33
Q32PX7	FUBP1_RAT	*Fubp1*	Far upstream element-binding protein 1	Nc	down	0.0283	-	-	1.78	5	97	10	67,326	7.18
Q794E4	HNRPF_RAT	*Hnrnpf*	Heterogeneous nuclear ribonucleoprotein F	Nc, Np	up	0.0105	-	-	0.11	4	158	21	46,043	5.31
Q6AY09	HNRH2_RAT	*Hnrnph2*	Heterogeneous nuclear ribonucleoprotein H2	Nc, Np	-	-	up	0.0285	0.42	5	194	28	49,547	5.89
P85973	PNPH_RAT	*Pnp*	Purine nucleoside phosphorylase	Cp	up	0.0550	-	-	0.59	6	263	31	32,566	6.46
P62716	PP2AB_RAT	*Ppp2cb*	Serine/threonine-protein phosphatase 2A catalytic subunit beta isoform	Cp, Nc, Cr	-	-	down	0.0143	2.41	4	169	16	36,123	5.21
P53042	PPP5_RAT	*Ppp5c*	Serine/threonine-protein phosphatase 5	Nc	down	0.0758	-	-	2.37	5	159	18	57,507	5.84
F1LPS8	F1LPS8_RAT	*Pura*	Transcriptional activator protein Pur-alpha	Nc, Cp	down	0.1093	down	0.0176	1.70	6	73	12	34,976	6.07
Q925G0	RBM3_RAT	*Rbm3*	Putative RNA-binding protein 3	Nc, Cp	down	0.0163	-	-	1.87	5	73	5	16,845	6.92
Q5XIG8	STRAP_RAT	*Strap*	Serine-threonine kinase receptor-associated protein	Cp, Nc	up	0.0163	up	0.0163	0.38	6	141	15	38,717	4.99
Q4KM49	SYYC_RAT	*Yars*	Tyrosine—tRNA ligase, cytoplasmic	Cp	up	0.0285	up	0.0547	0.57	5	141	18	59,420	6.57
P62961	YBOX1_RAT	*Ybx1*	Nuclease-sensitive element-binding protein 1	Cp, Nc, Cpg	up	0.0446	-	-	0.52	5	219	16	35,709	9.87
			**Signal Transduction**											
P97697	IMPA1_RAT	*Impa1*	Inositol monophosphatase 1	Cp	up	0.3367	-	-	0.48	6	214	16	30,834	5.17
P62260	1433E_RAT	*Ywhae*	14-3-3 protein epsilon	Cp, Mel	up	0.3367	-	-	0.51	6	213	34	29,326	4.63
			**Regulation**											
Q64640	ADK_RAT	*Adk*	Adenosine kinase	Cts, Nc	down	0.0039	-	-	4.50	6	129	13	40,450	5.72
Q02589	ADPRH_RAT	*Adprh*	(Protein ADP-ribosylarginine) hydrolase	Cp	up	0.0176	-	-	0.31	5	111	12	40,220	5.62
Q07936	ANXA2_RAT	*Anxa2*	Annexin A2	M, Mel	up	0.0782	-	-	0.53	6	71	12	38,939	7.55
P27139	CAH2_RAT	*Ca2*	Carbonic anhydrase 2	Cp	down	0.0104	-	-	5.01	6	89	11	29,267	6.89
P45592	COF1_RAT	*Cfl1*	Cofilin-1	Nm, Cp	up	0.1745	-	-	0.56	5	154	16	18,749	8.22
P08082	CLCB_RAT	*Cltb*	Clathrin light chain B	Cpv, M	down	0.0209	down	0.0209	2.31	4	104	8	25,216	4.56
B0BNE5	ESTD_RAT	*Esd*	*S*-formylglutathione hydrolase	Cp, Cpv	down	0.1003	-	-	1.86	5	181	15	31,971	6.44
P07936	NEUM_RAT	*Gap43*	Neuromodulin	M	up	0.0039	-	-	0.11	6	95	7	23,703	4.61
P50399	GDIB_RAT	*Gdi2*	Rab GDP dissociation inhibitor beta	Cp, M	up	0.1093	-	-	0.52	6	311	38	51,018	5.93
Q63228	GMFB_RAT	*Gmfb*	Glia maturation factor beta	Cp	up	0.0039	-	-	0.13	6	78	6	16,897	5.32
P62749	HPCL1_RAT	*Hpcal1*	Hippocalcin-like protein 1	Pem	down	0.0374	-	-	1.96	6	202	17	22,438	5.32
O88767	PARK7_RAT	*Park7*	Protein DJ-1	Cp, Nc, Mito	down	0.1093	-	-	1.74	6	280	21	20,190	6.32
Q1RP74	Q1RP74_RAT	*Tbcb*	Tubulin folding cofactor B	Nc, Cp, MT	up	0.0176	-	-	0.32	5	196	17	27,515	4.93
P70566	TMOD2_RAT	*Tmod2*	Tropomodulin-2	Cp, Csk	down	0.0250	-	-	3.39	6	185	15	39,468	5.34
P63029	TCTP_RAT	*Tpt1*	Translationally-controlled tumor protein	Cp	up	0.0106	-	-	0.29	5	135	10	19,564	4.76
P11232	THIO_RAT	*Txn*	Thioredoxin	Nc, Cp, Sc	up	0.0039	-	-	0.24	6	67	4	12,008	4.8
Q920J4	TXNL1_RAT	*Txnl1*	Thioredoxin-like protein 1	Cp	up	0.0105	-	-	0.19	4	257	19	32,628	4.84
			**Chaperones**											
Q6P502	TCPG_RAT	*Cct3*	T-complex protein 1 subunit gamma	Cp	-	-	up	0.0062	0.33	5	210	31	61,179	6.23
Q68FQ0	TCPE_RAT	*Cct5*	T-complex protein 1 subunit epsilon	Cp, Cts	up	0.0176	up	0.0330	0.31	5	329	43	59,955	5.51
P34058	HS90B_RAT	*Hsp90ab1*	Heat shock protein HSP 90-beta	Cp, Mel	down	0.2733	down	0.0104	2.39	6	260	42	83,571	4.97
P06761	GRP78_RAT	*Hspa5*	Heat shock 70kDa protein 5	ER, Mel	down	0.8728	down	0.0105	3.49	6	315	34	72,474	5.07
P11598	PDIA3_RAT	*Pdia3*	Protein disulfide-isomerase A3	ER, Mel	down	1.0000	down	0.9168	1.79	5	73	8	57,044	5.88
			**Structural Proteins**											
B2RYJ7	B2RYJ7_RAT	*Actr1b*	ARP1 actin-related protein 1 homolog B (Yeast)	Cp, Csk	down	0.0782	-	-	1.77	6	173	18	42,369	5.98
Q4V7C7	ARP3_RAT	*Actr3*	Actin-related protein 3	Cp, Csk	up	0.0163	-	-	0.52	6	230	27	47,783	5.61
B2GUZ5	CAZA1_RAT	*Capza1*	F-actin-capping protein subunit alpha-1	Cp, Csk	up	0.0250	-	-	0.57	6	230	19	33,060	5.43
Q5XI32	CAPZB_RAT	*Capzb*	F-actin-capping protein subunit beta	Cp, Csk	up	0.0163	-	-	0.57	6	184	20	30,952	5.69
P47819	GFAP_RAT	*Gfap*	Glial fibrillary acidic protein	Cp	down	0.0039	-	-	5.72	6	381	48	49,984	5.35
D4A6B2	D4A6B2_RAT	*Immt*	Mitochondrial inner membrane protein	Mito, Mim	down	0.0547	-	-	2.58	6	375	46	83,104	5.34
P23565	AINX_RAT	*Ina*	Alpha-internexin	Nf	down	0.0547	-	-	1.92	6	391	45	56,253	5.2
P30009	MARCS_RAT	*Marcks*	Myristoylated alanine-rich C-kinase substrate	Cp, M, La	up	0.0065	-	-	0.40	6	109	9	29,834	4.32
P19527	NFL_RAT	*Nefl*	Neurofilament light polypeptide	Nf, A	down	0.0039	-	-	3.49	6	412	53	61,355	4.63
P12839	NFM_RAT	*Nefm*	Neurofilament medium polypeptide	Nf, Csk, A	down	0.0090	-	-	2.84	5	291	48	95,848	4.77
B3GNI6	SEP11_RAT	*Sept11*	Septin-11	Cp, A	down	0.0163	down	0.25059	1.87	5	69	11	50,005	6.24
P13668	STMN1_RAT	*Stmn1*	Stathmin	Cp, Csk	up	0.3367	-	-	0.04	6	171	14	17,278	5.76
Q6AYZ1	TBA1C_RAT	*Tuba1c*	Tubulin alpha-1C chain	Cp, Csk	down	0.0190	-	-	3.73	4	87	8	50,590	4.96
Q3KRE8	TBB2B_RAT	*Tubb2b*	Tubulin beta-2B chain	Cp, Csk	up	0.3472	-	-	0.48	5	317	42	50,377	4.78
			**Transport Proteins**											
Q5M7T6	Q5M7T6_RAT	*Atp6v0d1*	ATPase, H+ transporting, lysosomal 38 kDa, V0 subunit d1	M	up	0.0039	-	-	0.23	5	111	11	40,731	4.89
Q811Q2	CLIC6_RAT	*Clic6*	Chloride intracellular channel protein 6	Cp, M	down	0.0039	down	0.01902	2.43	6	204	19	64,990	4.29
Q6AYH5	DCTN2_RAT	*Dctn2*	Dynactin subunit 2	Cp, M	up	0.0833	-	-	0.49	4	218	21	44,235	5.14
Q62871	DC1I2_RAT	*Dync1i2*	Cytoplasmic dynein 1 intermediate chain 2	Cp, Csk	down	0.1003	down	0.06789	2.13	5	111	11	71,533	5.11
P55051	FABP7_RAT	*Fabp7*	Fatty acid-binding protein, brain	Cp	up	0.0039	-	-	0.21	6	152	12	15,140	5.46
Q9Z2L0	VDAC1_RAT	*Vdac1*	Voltage-dependent anion-selective channel protein 1	Mom, M	down	0.0500	down	0.52243	2.44	4	215	17	30,851	8.62
P81155	VDAC2_RAT	*Vdac2*	Voltage-dependent anion-selective channel protein 2	Mom	down	0.0039	-	-	2.25	6	168	15	32,353	7.44

At developmental stage P7, the proteins involved in biosynthesis sum up to an amount of 23 proteins which are differentially expressed, from which 15 proteins show an up-regulation with respect to their expression in P90. The protein UMP-CMP kinase (Cmpk1) is required for cellular nucleic acid biosynthesis. Another up-regulated protein is aspartate-tRNA ligase (Dars)* which is a part of the multi-enzyme complex of aminoacyl-tRNA synthetases. Furthermore, elongation factor 1-alpha 1 (Eef1a1) and elongation factor 1-gamma (Eef1g) also belong to the group of up-regulated proteins at this stage of development. Both of them present subunits of the elongation factor-1 complex. Altogether, five proteins including the far upstream element-binding protein 1 (Fubp1) with an ATP-dependent DNA helicase function and the transcriptional activator protein Pur-alpha (Pura) are down-regulated at P7. In addition, three proteins are absent at P90 compared to P7 (for example, the protein eukaryotic translation initiation factor 4A1 (Eif4a1)*). Serine/arginine-rich splicing factor 1 (Srsf1)* plays a role in preventing exon skipping, ensuring the accuracy of splicing and regulating alternative splicing. Nuclease-sensitive element-binding protein 1 (Ybx1) mediates pre-mRNA alternative splicing regulation. The 60S acidic ribosomal protein P0 (Rplp0)* catalyzes protein synthesis and is a component of the 60S subunit and the L10P family of ribosomal proteins.

Ten regulatory proteins are down-regulated when comparing P7 to P90, like the protein adenosinkinase (Adk), for example. The protein tropomodulin-2 (Tmod2) presents a neuronal-specific member of the tropomodulin family of actin-regulatory proteins. Another neuron specific down-regulated protein is hippocalcin-like protein 1 (Hpcal1), a member of the calcium-binding proteins family found in the retina and brain. Also the protein DJ-1 (Park7)*, which acts as a positive regulator of androgen receptor-dependent transcription, shows a down-regulation. Also, 13 proteins altogether are up-regulated towards P90. The protein cofilin-1 (Cfl1), for example, which exhibits pH-sensitive F-actin depolymerizing activity and plays a role in the regulation of cell morphology and cytoskeletal organization. Neuromodulin (Gap43) is another up-regulated protein, typically expressed at high levels in neuronal growth cones during development and axonal regeneration. Tubulin folding cofactor B (Tbcb), another up-regulated protein, has the ability to bind to alpha-tubulin folding intermediates and is also involved in the regulation of tubulin heterodimer dissociation. Thioredoxin (Txn) and thioredoxin-like protein 1 (Txnl1) which also show an up-regulation towards P90 are both involved in different redox reactions and in the reversible *S*-nitrosylation of cysteines in certain proteins. An absent protein at this developmental stage, endophilin-B1 (Sh3glb1)* may be involved in regulating apoptotic signaling pathways and maintaining mitochondrial morphology.

The amount of up- and down-regulation of the structural proteins is more or less balanced. Eight proteins are up-regulated at this stage. These proteins are mainly involved in the dynamic organization of the cytoskeleton, like actin-related protein 3 (Actr3), member of the Arp2/3 complex, dihydropyrimidinase-related protein 3 (Dpysl3) or F-actin-capping protein subunit alpha-1 (Capza1), F-actin-capping protein subunit beta (Capzb) and Fascin (Fscn1)*. Other up-regulated proteins are the myristoylated alanine-rich C-kinase substrate (Marcks) which supports the crosslinking of actin filaments. Stathmin (Stmn1) also displays an up-regulation and is involved in the regulation of the microtubule filament system. Twelve proteins are down-regulated towards P90. These are mainly members of the septin family (septin-11 (Sept11), septin-5 (Sept5)*, septin-8 (Sept8)*) that are involved in a variety of cellular functions including cytokinesis and vesicle trafficking. Other down-regulated proteins are for example members of the neurofilament family (neurofilament light polypeptide (Nefl), neurofilament medium polypeptide (Nefm), alpha-internexin (Ina)) which comprise the axoskeleton and functionally maintain the neuronal caliber.

All in all, at developmental stage P7, a total of 140 proteins is expressed differentially as compared to P90, of which 63 proteins are up-regulated, 72 proteins are down-regulated and five proteins are absent at P90 (Table 2).

**Table 2:** Overview of the differential expression of the proteins in P7 *vs*. P90 as well as P637 *vs*. P90.

Categories	Amount (P7)	Amount (P637)
	(Absolute Numbers)	
Total	140	61
Up-regulated	63	30
Down-regulated	72	30
Absent	0	0
Absent P90	5	1

For P637 in comparison to P90, differences in the expression are also noticeable. At first, beginning with the functional group with the highest number of differentially expressed members, the proteins of the carbohydrate metabolism, nine proteins are up-regulated and one protein is down-regulated compared to P90. The up-regulated protein faction contains alcohol dehydrogenase (Akr1a1)*. This protein also shows an up-regulation at P7 towards P90. Fructose-bisphosphate aldolase C*, a member of the class I fructose-biphosphate aldolase gene family is expressed specifically in the hippocampus and Purkinje cells of the brain. A down-regulation of it can be seen at P7. Dihydrolipoyllysine-residue acetyltransferase component of pyruvate dehydrogenase complex (Dlat) also displays an up-regulation in this developmental stage. The down-regulated protein towards P90 of this category is phosphoglycerate kinase 1 (Pgk1)*. An expression change of it in P7 towards P90 could not be observed.

Four chaperone proteins are up-regulated. These contain two different subunits of the T-complex protein 1 (which has a molecular chaperone function), subunit gamma (Cct3) and epsilon (Cct5). Other up-regulated proteins include the endoplasmic reticulum resident protein 29 (Erp29)* which plays an important role in the processing of secretory proteins within the endoplasmic reticulum. Also the heat shock cognate 71 kDa protein (Hspa8)* is up-regulated at this stage which binds to nascent polypeptides to facilitate correct folding for example. However, also the down-regulated proteins (a total of five proteins) contain different members of the heat shock protein family (heat shock protein Hsp 90-beta (Hsp90ab1), heat shock 70 kDa protein 4 (Hspa4)*, heat shock 70kDa protein 5 (Hspa5). These proteins also show a down-regulation at P7 towards P90. Other proteins which are down-regulated are calreticulin (Calr)* and protein disulfide-isomerase A3 (Pdia3). While Calr presents a calcium-binding chaperone, Pdia3 interacts with lectin chaperones calreticulin a calnexin to modulate folding of newly synthesized glycoproteins.

The proteins which are involved in the pathways of the protein biosynthesis consist of four proteins which are up-regulated and five proteins which display a down-regulation at P637. The up-regulated group contains the heterogeneous nuclear ribonucleoprotein H2 (Hnrnph2), a component of the heterogeneous nuclear ribonucleoprotein complexes. In addition, the delta interacting protein 2 (Poldip2)* also shows an up-regulation at this developmental stage. The group of down-regulated proteins includes for example serine/threonine-protein phosphatase 2A catalytic subunit beta isoform (Ppp2cb) as well as the transcriptional activator protein Pur-alpha (Pura) which plays a role in the initiation of DNA replication and recombination.

At P637, a total amount of 61 proteins are expressed differentially as compared to P90, of which 30 proteins show an up-regulation, 30 proteins show a down-regulation and one protein is absent in P90 (Table 2).

## 3. Discussion

In general, the Ce does not contain a homogeneous cell population. For this reason, a differential proteome analysis includes not only the proteome changes of the neuronal populations, but also the differential expression of proteins from other cell populations in the Ce. The technique of separation used in this study, allows a separation within the range of approximately 10–100 kDa and between a pH-range of 3–10. Therefore, it is possible to analyze a major part, but not the entire proteome of the Ce [22]. In addition, the external parts of the gel show a lower resolution which presents another possibility for a misinterpretation of the regulation analysis of some protein spots. Additionally, it is known that proteins may be compromised in multiple spots within different locations in the gel which can be a reason for different posttranslational modifications and isoforms of the same protein. Therefore, different proteins within a particular spot may show only an average change of the spot volume, but this does not concurrently apply for a specific protein of such a mixture of proteins within the same spot (see Table S1, Figure S2). With the aid of other mass spectrometric detection methods like the stable isotope ratio mass spectrometry (SIRMS), it would be possible to identify both the protein and its variants more precisely and to overcome the sample-to-sample recovery variabilities associated with non-SIRMS MS-proteomic methods. In addition, an improved analysis of the lower expressed and abundant proteins would be allowed by using affinity-enrichment-MS methods and targeted biomarker discovery applications (e.g., IDBEST™, iTRAQ™) [23]. Therefore, the results of this study dealing with the proteomic analysis of the Ce should be seen as a first step towards getting an overview of the differentially expressed protein patterns in this region of the brain.

This study focuses on the analysis of expression differences in the abundance of proteins and gives an overview of developmental changes in the rat Ce by analyzing snapshots (P7, P90, P637) in the development. Focus is placed on differences in the expression of proteins for neuronal development in the Ce. Changes in the expression of proteins were analyzed and compared with existing findings in the literature. Specific changes of cytoplasmatic proteins of the postnatal developing Ce were found. However, these proteins need to be validated by Western-Blots and/or immunohistochemistry in an ongoing study. Such a verification is also important with regard to some differences in statistically significant and not significant changes of protein expressions by applying the multi condition coverage method and the Mann-Whitney *U*-test.

At the developmental stage P7 in comparison to P90, especially proteins involved in biosynthesis, regulatory proteins and structural proteins showed increased expression changes. At P637, mainly proteins of the carbohydrate metabolism, chaperones and proteins participating in the biosynthetic processes demonstrated the highest amount of differentially expressed proteins. These findings suggest that the above mentioned categories of proteins are important for the development, for example, for the maintenance of the cytoskeleton and the energy metabolism of the rat cerebellum. At P7, the majority of the differentially expressed proteins involved in regulatory processes are down-regulated when compared to P90. Several of the differentially expressed proteins of this category are associated with the regulatory dynamics of the cytoskeleton in the Ce as well as transmitter release. For example, the protein adenosinkinase (Adk), down-regulated at P7, presents a protein which is involved in three main processes [24]. It has the ability to influence the concentration of cyclic AMP and, therefore, acting as a chemical link between electrical activity and the changes in the intracellular concentration of cyclic AMP [25]. Secondly, it may also be involved in the vasodilatory regulation of cerebral blood flow in arterioles [26,27]. The alteration of the properties of synaptic transmission between parallel fibers and Purkinje cells presents another possible function of this protein [28]. Furthermore, Studer *et al.* [29] were able to show that in the early development, hippocampal adenosine kinase expression appears mainly in neurons and shifts to glia during the second postnatal week. Another down-regulated protein involved in the organization of the cytoskeleton is tropomodulin-2 (Tmod2). This protein, as mentioned in the results, is responsible for the regulation of actin filaments by capping the pointed ends of an actin filament and together with tropomyosin, stabilizing the filament and regulating its length [30,31]. Therefore, actin plays an important role in growth cone motility during development and the pre- and postsynaptic morphology of neurons. Especially Tmod2, also refers to as neuronal Tmod, is the isoform found in neuronal structures. Hippocalcin-like protein 1 (Hpcal1) is another protein down-regulated in the early stages of development in the Ce. This protein displays a high homology to hippocalcin which presents a neuron-specific calcium-binding protein, highly expressed in hippocampal pyramidal cells. This protein is able to interact with neuronal apoptosis inhibitory proteins to promote neuronal survival [32]. Its increase in expression in the developing hypothalamus suggests that Hpcal1 may promote neuronal survival in this region [33]. Other proteins involved in the organization of the cytoskeleton, cofilin-1 (Cfl1) and neuromodulin (Gap43) show both an up-regulated expression compared to P90. While Cfl1 is involved in the regulation of actin cytoskeleton dynamics and cell morphology, Gap43 is responsible for establishing neuronal growth cones formation during development and axonal regeneration. For Cfl1, a higher expression in the early stages of development agrees with the findings of Gurniak *et al*. [34] in which this protein is essential for the neuronal development in the mouse. For example, the establishment of neural crest cell migration and closure effects of the neural tube is impaired by the deletion of Cfl1 in the mouse. Console-Bram *et al*. [35] were able to determine that the expression of Gap43 mRNA in cerebellar granule cells and inferior olivary cells during development indicates that this growth-associated protein is initially involved in granule cell differentiation and migration, parallel and climbing fiber axonal outgrowth and synaptogenesis [36]. Also in their study, it could be shown by Northern blot analysis that cerebellar GAP-43 mRNA expression increases from birth to postnatal day 7 and then declines to a lower level in the adult. Especially in the deep cerebellar nuclei the protein was found to be most intense during the first postnatal week. This suggests that GAP-43 may influence Purkinje cell axon and cerebellar nuclear dendritic synaptogenesis as synaptic contact takes place during the first postnatal week [37,38]. The later decrease may indicate completion of axonal outgrowth from the deep cerebellar nuclei [35]. Also other proteins involved in the regulation of the cytoskeleton and neurogenesis show a difference in their expression during the development stages. Tubulin folding cofactor B (Tbcb), also up-regulated at P7, is located in the neurite ends of neuroblasts and in the growth cone transition zone which is essentially responsible for the negative regulation of axonal growth and microtubule dynamics [39]. Endophilin-B1 (Sh3glb1)* is absent at the early development stage of P7. Sh3glb1, a member of the BAR domain protein super-family, is an essential element of cellular traffic. It has a prominent function in synaptic vesicle endocytosis, receptor trafficking and apoptosis, and in other processes that require remodeling of the membrane structure [40]. There are different isoforms of this protein available, for endophilin-B1, this protein has been implicated in many stages of clathrin mediated synaptic vesicle endocytosis, from early events generating membrane curvature, to later stages such as vesicle fission and uncoating [41,42]. Furthermore, a selective depletion of endophilin from rat brain cytosol inhibited the generation of synaptic-like microvesicles and markedly reduced the formation of dynamin-coated tubules on synaptic membranes [41,43].

The majority of proteins involved in the processes of protein biosynthesis display an up-regulation at P7 in comparison to P90. Especially proteins involved in nucleic acid biosynthesis (Cmpk1) and the supply of new synthesized tRNA (Dars*, Eef1a1, Eef1g) are up-regulated. In the mammalian nervous system, transcripts exhibit multiple forms of splicing which presents an important step in growth processes such as axon guidance, synaptogenesis, and the regulation of membrane physiology [44,45,46,47]. Furthermore, different members of the family of the heterogeneous nuclear ribonucleoproteins (hnRNPs) are partially up- and down- regulated towards P90. While the heterogeneous nuclear ribonucleoprotein K (Hnrnpk)* and heterogeneous nuclear ribonucleoprotein F (Hnrnpf) show a greater expression at P7 compared to P90, other members (heterogeneous nuclear ribonucleoprotein H1, isoform CRA_b (Hnrph1)*, for example) present a higher expression at later stages (P637) of development. This protein family plays key roles in mRNA metabolism, DNA-related functions and microRNA biogenesis by regulating stability, localization and translation of a number of transcripts which represents an important step in the establishment and maintenance of nerve cell functions [48,49,50,51,52]. The regulation of the expression of many RNAs depends on the activity of RNA-binding proteins, often acting in concert and organized in complexes, probably regulating the activity and the expression of the other components of the complex itself [53]. The different expression levels of these members could indicate they are involved in different tasks and during development. For example, Hnrnpk* is also involved in other cellular processes, such as modulation, translation mRNA transport and signal transduction [54,55]. As Proepper *et al*. [56] were able to demonstrate Hnrnpk, plays an important role during neuronal differentiation and early synapse formation by interacting with other proteins which are important for dendritic branching and synapse formation (Abelson-interacting protein 1 (Abi-1), for example). 

As mentioned in the results, the amount of structural proteins shows a balanced amount of up- and down-regulated proteins in the analyzed developmental stages. Especially proteins for the dynamic organization of the cytoskeleton are up-regulated at the stage of P7. For example, different subunits of the F-actin-capping protein (F-actin-capping protein subunit alpha-1 (Capza1), F-actin-capping protein subunit beta (Capzb)) and additional proteins, important for the functional formation of actin and the cytoskeleton are up-regulated at this stage (stathmin (Stmn1), myristoylated alanine-rich C-kinase substrate (Marcks)). As Devaux *et al*. [57] stated, the ability to dynamically organize parts of the cytoskeleton can support neuronal differentiation, including axonal growth, branching and dendritic development. Therefore, an up-regulation of these proteins appears to be an important step for the cytoskeletal organization in the Ce. Further structural proteins are members of the neurofilament family (neurofilament light polypeptide (Nefl), neurofilament medium polypeptide (Nefm)) which are functionally related to axonal growth, neuronal polarity and signaling in axon guidance [58] are down-regulated at P7 in comparison to P90. As mentioned by Riederer *et al*. [59], in the Ce, changes in the expression and distribution of the neurofilament proteins appear during maturation differentially for different cell types. For example, in the Ce of kittens, while afferent mossy and climbing fibers in the medullary layer contained NF-M and NF-L already at birth, other cell types show a different expression of these proteins. Within the first three postnatal weeks, for example, all three subunits appeared in mossy and climbing fibers in the internal granular and molecular layers and in the axons of Purkinje cells. Axons of local circuit neurons such as basket cells expressed these proteins at the end of the first month, whereas parallel fibers expressed them last, at the beginning of the third postnatal month. Also different members of the septin-protein family are down-regulated at P7. Also these proteins seem to have an important role in the developing brain by being integrated in microcircuits, including the dendritic arborization and the synthesis of dendritic spines. While septin 5 (Sept5)* is localized in the presynaptic terminals and frequently associated with synaptic vesicles [60,61,62], septin 11 (Sept11) also plays a role in GABAergic synaptic connectivity, particularly within the postsynapse, and concentrates at the neck of dendritic spines in the intact brain [63]. A differential protein expression was also observable in aged rat brains (P637). The main differences in the expression concern the categories of proteins of the carbohydrate metabolism, proteins of the biosynthesis and chaperones.

The proteins which are functionally integrated in the glucose metabolism clearly demonstrate a high amount of up-regulated proteins towards P90. As described in the results, except for one protein (phosphoglycerate kinase 1 (Pgk1)*), all other proteins show an up-regulation in expression at this developmental stage. Several of these proteins are involved in the utilization of glucose by being integrated or associated in the tricarboxylic cycle (e.g., dihydrolipoyllysine-residue acetyltransferase component of pyruvate dehydrogenase complex (Dlat)). Also Vannucci R.C. and Vannucci S.J. [64] were able to demonstrate that the utilization of glucose to maintain tissue energy stores is increased in the newborn rat brain. However, the glucose transport capacity and simultaneously the rate of glucose uptake of the immature brain are much lower than in the adult animal. Under normoxic conditions, glucose uptake into newborn rat brain proceeds at one-fifth the rate it does in adult brain [65]. Furthermore, it is known that with increasing age, a decrement of the cerebellar glucose metabolic rate can be observed which also reflects significant impairments in several cognitive tasks for example. An additional increase in the expression of proteins which are involved in the glucose metabolism between the adult age (P90) and aged rats (P637) in our study could, therefore, be an indicator for the reaction of the animal to the decreasing metabolic rate to maintain a physiological state of glucose metabolism conditions and to supply energy for neuronal integrity for example. 

The proteins involved in different tasks of protein biosynthesis belong to the group which displays the second highest rate of differentially expressed proteins in the Ce of aged rats compared to adult animals. Here, the amount of up- and down-regulated proteins is balanced at this stage. As mentioned in the results, the protein heterogeneous nuclear ribonucleoprotein H2 (Hnrnph2) is one of the members of the up-regulated group, a protein important for serving as a crucial checkpoint in pre-mRNA processing [66]. In general, members of this protein family (heterogeneous nuclear ribonucleoprotein complex) are involved in mRNA metabolism, DNA-related functions and microRNA biogenesis, including processing in alternative splicing during neuronal differentiation [48]. These proteins include a diverse group of proteins containing RNA binding motifs as well as several auxiliary domains. Therefore, a simultaneous binding of pre-mRNA and other proteins is possible [67]. As Meshorer *et al*. [66] describes in his work, a misregulation of these proteins by aging can influence splice site selection or lead to alternative or aberrant splicing of different downstream target sequences which then can lead to uncontrolled cell proliferation or to different age-related diseases (Age-related macular degeneration, Alzheimer’s disease, *etc.*). As stated by Tollervey *et al.* [68], especially genes with metabolic functions show expression changes during aging which are linked with alternative splicing. This indicates that alternative splicing complements the transcriptional regulation in modifying the molecular machinery for the repair of oxidative DNA damage in the brain [69]. The group of down-regulated proteins contains the protein serine/threonine-protein phosphatase 2A catalytic subunit beta isoform (Ppp2cb). As described in the results, this protein is responsible for regulating the negative control of cell growth and division, for example. As mentioned by Price and Mumby [70], protein phosphatase 2A can also have unique functions in neuronal cells. Within neurons, PP2A seems to be targeted to specific intracellular locations, such as neurofilaments by probably controlling stability and interactions with other components of the neuronal cytoskeleton [71]. This indicates that at this developmental stage, also the amount of expressed neurofilaments should decrease. At least in our study a change of expression between P90 and P637 was not observable. However, Vega *et al*. [72] were able to show that in old rats a significant decrease in the density of neurofilament light polypeptide immnoreactivity occurs, suggesting that cytoskeletal abnormalities are also present within physiological aging.

Moreover, the category of chaperones also displays an amount of proteins which have a differential expression in the analyzed developmental stages. The up-regulated proteins consist of two different subunits of the T-complex protein 1, subunit gamma (Cct3) and epsilon (Cct5) members of the chaperonin containing TCP1 complex. Actin and tubulin are consistently found in the TCP-1 complex favoring the idea that they are its major substrates [73,74]. The up-regulation of these proteins could be an indication of increasing stress induced by aging and possibly a misregulation of the cytoskeletal portion in the Ce. As described by Proctor *et al*. [75], neurodegeneration is an age-related disorder which is characterized by the accumulation of aggregated protein, loss of protein homeostasis and neuronal cell death. The capacity of chaperones is sufficient to maintain protein homeostasis. Even under conditions of increasing stress with age, normal chaperone capacity is able to process the increasing overload due to the mechanism of up-regulation of chaperones after stress. Though other chaperones (heat shock protein Hsp 90-beta (Hsp90ab1), heat shock 70 kDa protein 4 (Hspa4)*, heat shock 70 kDa protein 5 (Hspa5)) show a down-regulated expression at this stage. As stated by Soerensen and Leoschke [76], a possible theory for the decreased expression of these proteins could be an adaptive down-regulation of the energetically expensive Hsp-system to conserve energy for other purposes, directly causing the decrease in stress resistance with age. Another reason for the decreased expression of proteins with chaperone and folding functions could indicate a loss of protein quality regulation with aging that may contribute to the buildup of cytoskeletal proteins (e.g., increased neurofilament light chain and tubulin) with increasing age [77].

In addition, it could be determined that other differentially expressed proteins of the analyzed developmental stages are involved in proliferation, migration and differentiation. For example, proteins participating in transport processes, additional proteins involved in the carbohydrate metabolism and proteins involved in the energy homeostasis of the brain indicate additional important protein categories for the development of the Ce. Also Becker *et al*. [78] were able to find regional differences in the Ce proteome in comparison to the inferior colliculus, for example. In this study, especially Proteins involved in energy metabolism including proteins participating in glycolysis, intracellular signaling cascades and vesicle trafficking showed main differences in expression. Others [79] were able to identify changes in the protein expression in brains of different ages. Also here, they were able to provide evidence for the differential regulation of specific proteins which are important for several metabolic pathways including regulatory proteins.

Further investigation is needed, but some of these proteins could present important factors for the differentiation and survival of transplanted progenitor cells in neonatal and adult striata. In additional studies, this could also open up the possibility to associate the differential expressed proteins to their specific metabolic pathways and to determine the factors which are important for the differentiation of neuronal progenitor cells. Furthermore, in the brains of adult (P90) and aged rats (P637) a differential expression was also detectable. This indicates that in the adult Ce, there are still remodeling processes for different categories of proteins possible ongoing which could play a role in cell replacement by aging, learning and memory as well as synaptic signaling and messaging. This also offers the option to analyze and separate proteins to determine factors which show an importance in the protein expression, for other major cerebellar disorders like NPC1 for example, in future studies.

## 4. Experimental Section 

### 4.1. Treatment

Male Wistar rats (Rattus norvegicus, Charles River, Sulzfeld, Germany) of different ages (7, 90, 637 postnatal days) with six animals in each group were used for this study [80,81]. The animals were housed at 22 ± 2 °C under an artificial day and night rhythm with a 12 h light–dark cycle with free access to water and standard nutrition. The animal treatment and experimental procedures were conducted in compliance with the regulations and licensing of the local authorities (Landesamt für Landwirtschaft, Lebensmittelsicherheit and Fischerei Mecklenburg Vorpommern, Germany) and the Animal Care and Use Committee of the University of Rostock. According to the European Communities Council Directive of 24 November 1986 (86/609/EEC) and in accordance with the above-mentioned local authorities adequate measures were taken to minimize pain or discomfort.

### 4.2. Perfusion and Dissection

At defined dates (7, 90, 637 days postnatal), perfusion was performed. The animals were anesthetized with ether and killed by intraperitoneal Pentobarbital-Na^+^-injection (60 mg/kg BW). Transcardial perfusion was performed with 100–400 mL (bodyweight depending) cooled (4 °C) 0.9% NaCl-solution. After decapitation and brain dissection, the dissected brain regions were weighed and stored at −80 °C until homogenization. From pentobarbital injection to −80 °C storage it took less than 5 min.

### 4.3. Homogenization

The extraction of proteins was performed according to published standardized protocols [82,83,84]. Each developmental stage (P7, P90, P637) consisted of six gel images. For P7, P90 and P637, each gel image presents a single dissected brain region. The brain sections were incubated with (9× probe mass (mg)) μL lysis buffer consisting of 7 M urea (Sigma, Steinheim, Germany), 2 M thiourea (Sigma), 4% CHAPS (Sigma), 70 mM DTT (Sigma), 0.5% Bio-Lyte Ampholytes pH 3–10 (Fluka, Buchs, Switzerland) and a mixture of protease inhibitors (Roche, Basel, Switzerland) additionally enriched with (0.1× probe mass (mg)) μL Pepstatin A and PMSF (Fluka) and snap-frozen at −150 °C. The samples were quickly thawed and transferred into a 2 mL Wheaton potter (neo-lab, Heidelberg, Germany) for homogenization. In the next step, glass beads (Roth, Karlsruhe, Germany) were added to the suspension, following a 15 s sonication, 15 s vortexing (repeated six times) and finished by shock freezing the suspension at 150 °C. After quickly thawing the samples, they were put in a beaker on a magnetic stirrer that was filled with ice water for 15 min. Finally, the samples were centrifuged at 17,860× *g* for 20 min at 4 °C. The supernatant was very carefully removed using a 2 mL syringe (Becton Dickinson, Heidelberg, Germany) with a 0.5 × 25 mm needle (Becton Dickinson), because of a thick lipid coverage derived from myelinated nerve fibers. The protein concentration of the supernatant was determined by the Bradford assay.

### 4.4. Two Dimensional Polyacrylamide Gel Electrophoresis (2DE)

#### 4.4.1. Rehydration

The first dimension was performed in a PROTEAN IEF cell system (Bio-Rad, Berkeley, CA, USA). Protein extracts of 1 mg protein were loaded on immobilized pH 3–10 nonlinear gradient strips with a length of 17 cm (GE-Healthcare, Buckinghamshire, UK; Art.: 17-1235-01) and actively rehydrated with 300 μL rehydration buffer consisting of 6 M urea (Sigma), 2 M thiourea (Sigma), 2% CHAPS (Sigma), 16 mM DTT (Sigma), 0.5% Bio-Lyte Ampholytes pH 3–10 (Fluka) at 50 V for 12 h at 20 °C.

#### 4.4.2. First Dimension: Isoelectric Focusing

After rehydration, to reduce artifacts, electrode wicks (Bio-Rad) were added. Focusing started with the “conditioning step” (2 h) which subdivides in two sub-steps: (a) linear voltage rise to 500 V, step-hold 30 min; (b) linear voltage rise to 2500 V, step-hold 1 h. After that, the “slow voltage ramping” (2.5 h): quadratic voltage rise to 8000 V and the “final focusing”: actual process of focusing (duration: 50.000 Vhrs) were performed. During the whole IEF the temperature was constantly kept at 20 °C. After focusing the strips were stored at −80 °C.

#### 4.4.3. Second Dimension: Polyacrylamide Gel Electrophoresis

Focused IPG-strips were equilibrated in two steps of 30 min each in 5 mL of freshly prepared SDS equilibration solution consisting of 1.5 M Tris–HCl pH 8.8 (Roth), 6 M urea (Sigma), 30% glycerol (Sigma), 2% SDS (Sigma), trace of bromophenol blue (Roth) supplemented with 10 mg/mL DTT and 40 mg/mL iodoacetamide.

The strips were transferred on 12% homogeneous self-cast sodium dodecyl sulfate polyacrylamide gels (200 mm × 250 mm × 1.5 mm). At 125 V per gel (Power Pac 1000, Bio-Rad), they were run in the PROTEAN Plus Dodeca Cell (Bio-Rad). To ensure a constant buffer-temperature of 10 °C, a cooling device (Julabo F10, Julabo Labortechnik, Seelbach, Germany) was used.

#### 4.4.4. Fixation and Staining

Fixation was performed with acetic acid-methanol solution (45% methanol, 1% acetic acid overnight. Staining of the gels was performed in a colloidal CBB G250 solution (1 g/1000 mL) (Roth) as described previously [85]. After 24 h, the gels were destained with ultrapure water and were held in cold storage (4 °C) with ultrapure water until digitization.

### 4.5. Gel Analysis

#### 4.5.1. Digitization

The stained gels (*n* = 6) were scanned as 12 bit gray scale tif-images with a F4100 scanner (Heidelberg, Heidelberg, Germany) at 300 dpi resolution. Gels were rinsed in 0.02% sodium azide (Aldrich Chemie, Steinheim, Germany), shrink-wrapped in plastic and stored at 4 °C until picking for MALDI-TOF-MS.

#### 4.5.2. Digital Gel Processing

For 2DE-gel image analysis, the software package Progenesis PG200 Version 2006 (Nonlinear Dynamics Ltd., Newcastle upon Tyne, UK) was used. The gels were registered to a reference gel (the gel which contained most spots with highest separation and staining quality and least artifacts) and manually edited spots were matched to allow comparability of all gels (Figure 1).

#### 4.5.3. Determination of Differentially Abundant Protein Spots

Protein spots in 2DE were quantified by normalizing spot volumes using the Progenesis PG200 (Nonlinear Dynamics Ltd., Newcastle upon Tyne, UK) and spot volume differences were calculated. After the comparison of the normalized gray value-spot volumes of all generated spot pairs with Access and Excel (Windows, Microsoft Corporation, Redmond, WA, USA), the comparison of the spot volumes was determined by calculation of the spot volume quotient (SVQ, P90/P7 or P90/P637). Only those spots were considered to be significantly up- or down-regulated that showed a SVQ (Spot Volume Quotient) of 0.6 or less and 1.67 or greater [83,84,86]. The differences were evaluated significantly if differentially expressed spots were detected in at least four gel images (correlated spots) belonging to one test group. Instead of applying a parametric or non-parametric statistical test, the multi condition coverage method was used with condition 1: change of SVQ < 0.67 or SVQ > 1.67 and condition 2: changes in four of six gels. In addition the nonparametric Mann-Whitney *U*-test was applied. If multiple spots of one protein were detected by mass spectrometric analysis, the differential expression was determined by the mean of their individual expression levels. Additionally, each protein which exists in a mixed spot with at least another protein in one developmental stage was marked individually as well as if one protein was present in multiple spots per stage (see Table S1). The classification of the differentially expressed proteins in their respective functional protein groups itself was generated by a comparison of the proteins’ properties; in addition, the basic function of each protein is briefly described (according to Entrez Gene, GeneCards, UniProtKB/Swiss-Prot, and/or UniProtKB/TrEMBL).

#### 4.5.4. Mass Spectrometric Analysis of Protein Spots

According to published standardized protocols [87], protein spots were excised from the gels with a spot picker (Flexys Proteomics picker, Genomic Solutions, Ann Arbor, MI, USA), transferred into 96-well plates, and subjected to in-gel digestion with trypsin. The gel plugs were washed twice with 30% acetonitrile (ACN) in 25 mM ammonium bicarbonate and 50% ACN in 10 mM ammonium bicarbonate, respectively, shrunk with ACN, and dried at 37 °C. The dried gel plugs were re-swollen with 5 μL protease solution (sequencing grade trypsin, 10 ng/μL in 3 mM Tris–HCl, pH 8.5, Promega, Madison, WI, USA) and incubated for 8 h at 37 °C. Thereafter, 5 μL of extraction solution (0.3% trifluoroacetic acid, 50% ACN) were added and the samples were agitated at room temperature for 30–60 min before the peptide extracts were transferred into the 96-well collection plates. The resulting peptide-containing solution was prepared for MALDI analysis by spotting 0.6 μL of the tryptic digest and 0.45 μL of matrix solution consisting of 9 mg/mL α-cyano-4-hydroxy-cinnamic acid (CHCA) in 50% ACN, 0.1% trifluoroacetic acid on standard stainless steel MALDI plates. MALDI-MS analysis was performed on a 4700 Proteomics Analyzer MALDI-TOF/TOF mass spectrometer (Applied Biosystems, Foster City, CA, USA). All acquired spectra were processed using 4700 Explore™ software (Applied Biosystems, Warrington, Cheshire, UK). For protein identification, spectra were submitted to MASCOT (version 2.4.0, Matrix Science, London, UK) via the MASCOT Deamon. Searches were performed against the subset of rat proteins of the UniProtKB protein sequence database (2012_01; 42755 sequences from Rattus). A mass tolerance of 60 ppm and one missing cleavage site were set, oxidation of methionine residues was considered as variable modification, and carbamidomethylation of cysteines as fixed modification. Peptide masses of trypsin autoproteolysis products and matrix-derived peaks were excluded. Identifications with Mascot scores greater than 59 were considered significant (*p* < 0.05). All results were examined carefully for reliability and occurrence of multiple proteins in the same sample.

## 5. Conclusions

In conclusion, the proteomic analysis of the Ce at three different developmental stages displays multiple changes in the expression of proteins. This includes a down- and up-regulation of single proteins and the occurrence of single proteins which are only present at specific developmental stages. Although the analysis of the differential 2D gels spot abundance does not exclude the simultaneous presence of proteins/isoforms with no detectable abundance differences. As mentioned in the results, the presence of abundance of single proteins could also result from different posttranslational modifications. However, the main focus of this study was to detect differences in the abundance of single protein spots to provide indications of developmental changes in protein expression during the development (P7, P90, P637) of the cerebellum and not in the characterization of structural differences of one protein in different spots, for example. As a further step, in an analysis of single protein candidates, all individual spots belonging to the protein have to be detected and its differential regulation has to be identified, including spots which show a lower resolution in the gel. Therefore, increasing the system’s sensibility and reproducibility by using additional methods (e.g., difference gel electrophoresis, DIGE), as well as the application of purification protocols could provide a more detailed view on small proteins (e.g., neurotransmitters, plasma membrane proteins) for further analysis. Another option would be an enhancement of the SVQ ranges (>1.67, <0.6) and the analysis of additional postnatal dates to give more insight in the development of the rat Ce. Though, an overview in the analysis of developmental protein expression changes in the Ce at the postnatal days of P7, P90 and P637 could be evaluated in this study.

## Supplementary Materials

Supplementary materials can be found at http://www.mdpi.com/1422-0067/16/09/21454/s1.

## Abbreviations

A: Axon
CBB: Coomassie blue
Cpg: Cytoplasmic granule
Cts: Centrosome
Cr: Chromosome
Csk: Cytoskeleton
cp: Chaperones
Cpm: Cytoplasm
Cpv: Cytoplasmic vesicle
Cts: Cytosol
ER: Endoplasmic reticulum
Eds: Endosome
Exr: Extracellular region
Gc: Growth cone
Gj: Gap junction
Golgi: Golgi apparatus
hGc: Heterotrimeric G-protein complex
La: Lipid-anchor
Lyso: Lysosome
M: Membrane
Micro: Microsome
Mmt: Mitochondrial matrix
Mm: Mitochondrial membrane
Mim: Mitochondrion inner membrane
MimS: Mitochondrion intermembrane space
Mom: Mitochondrion outer membrane
Mel: Melanosome
Mito: Mitochondrion
MT: Microtubule
Nc: Nucleus
Nf: Neurofilament
Nm: Nucleus matrix
Np: Nucleoplasm
P: Proteasome
pa: Proteins antioxidants
paam: Proteins amino acid metabolism
pb: Proteins biosynthesis
pcm: Proteins carbohydrate metabolism
pd: Proteins degradation
Per: Peroxisome
pem: Proteins energy metabolism
Pema: Proteinaceous extracellular matrix
pfm: Proteins fat metabolism
PMp: Peripheral Membrane protein
pr: Proteins regulation
pst: Proteins signal transduction
ptm: Proteins transmitter metabolism
Rs: Ribosome
S: Synapse
Sc: Secreted
Scc: Spliceosomal complex
sER: Smooth endoplasmic reticulum
sp: Structural proteins
Sv: Synaptic vesicle
Ss: Synaptosome
SR: Sarcoplasmic reticulum
TCA: Trichloroacetic acid
tp: Transport proteins
Ulc: Ubiquitin ligase complex
