# Supplementary material for: The Proteome Profiles of the Cerebellum of Juvenile, Adult and Aged Rats—An Ontogenetic Study
Source: Int J Mol Sci. 2015 Sep 7;16(9):21454–85. doi: 10.3390/ijms160921454 (PMC4613263; doi:10.3390/ijms160921454)
Supplement: Supplementary file 1 [file ijms-16-21454-s001.zip › ijms-90226-Supplementary Information/ijms-90226-Supplementary.pdf]

# Supplementary Information

In the following, an overview of the Ce ontogenesis regarding its cellular composition in detail is listed in the beginning (A, see Figure S1). Furthermore, the remaining differentially expressed proteins of the categories which are not listed in the result are presented. (B: Remaining categories of differentially expressed proteins of P7 in comparison to P90. C: Remaining categories of differentially expressed proteins of P637 in comparison to P90.)

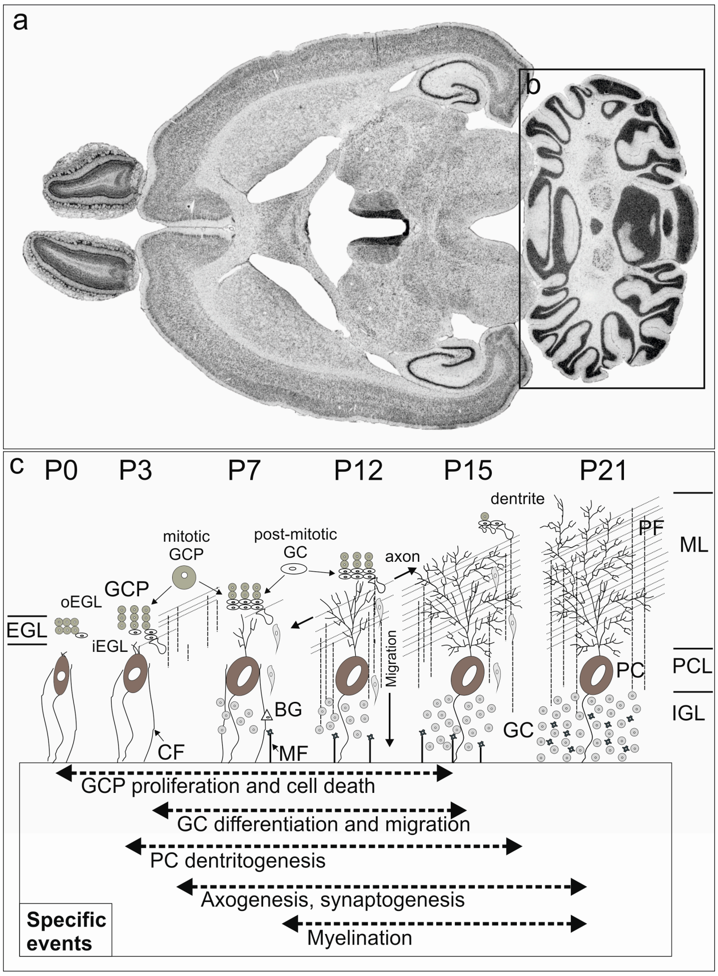


**Figure S1.** (**a**) Horizontal section of a rat brain; (**b**) Magnified view of the cerebellum (CE); (**c**) Schematic overview of the major stages of postnatal cerebellar cortex development. The granular cells (GCs) are generated by the proliferation of granule cell progenitors (GCPs) of the external granular layer (EGL) during the first and second week after birth. After the bilateral extension of parallel fiber (PF) axons by the post-mitotic GCs, the cell bodies migrate downwards through the developing molecular layer (ML), settling in the internal granular layer (IGL) below the Purkinje cell layer (PCL). The EGL is divided into two sub-layers: the outer EGL (oEGL) contains proliferating GPCs and the inner EGL (iEGL) containing post-mitotic GCs. Bergmann glia cells (BG) extending into the ML appear to guide GCs that reach the IGL. After a differentiation and additional extension of the dendrites into glomeruli, a connection of GCs to excitatory afferent mossy fibers (MFs) and inhibitory Golgi cell axons is possible. Purkinje cells (PCs) then have a robust outgrowth of their dendrites on which PCs receive two excitatory inputs from PFs and climbing fibers (CFs).

During the postnatal period of 3 weeks, this region is integrated into a functional circuit of the rat neuronal system [88].

As regards the postnatal development of the rat Ce, the main growth takes place immediately after birth. In the first 3 months, the mass of the Ce increases by a factor of 20.7. Up to the adolescence of the rat (P25–P60), no major increase or decrease of brain mass could be observed. As for the neuronal cells, an increase in the amount of cells to 150 million can be determined during birth and upon reaching adulthood. The addition of neurons occurs until P21 where at the same time a loss of neurons does not happen. Furthermore, the net number of neurons doubles till the adult age, while between P15 and P25 an increase of 5.9 mill cells happens. After a noticeable increase in non-neural cells in the whole rat brain between the second and third postnatal week, in the Ce, a loss of almost 50% of these cells can be observed between P17 and P25. After this process ends, a roughly 60% increase in these cells is remarkable [89].

In the first postnatal weeks, a significant migration of cells in the rat brain occurs. Because most of the neuronal migration is finished at the time of birth, these are mostly glial progenitor cells. Besides the genesis of interneurons of the olfactory bulb and migrating granular cells from the dentate gyrus to the hippocampus, also changes in the Ce after birth are observable. In the Ce, the generation of granular cells happens in the early postnatal development stage. Here, the outer granular cell layer of the brain surface produces the granular cell population by radial migration [90].

For the total cell amount in the rat brain, the Ce is the only region which shows an additional increase in the first week after the time of birth. Here, an additional growth of cells in the first postnatal week as well as after the third postnatal week can be found. Also, the size of the cells in the Ce and olfactory bulb are subject to ongoing changes. During neurogenesis, a decrease of the size of granular neurons is observable [91]. In this region, the excitatory neurons are generated by the granular progenitor cells in the external germinal layer during the first two postnatal weeks (see Figure S1). The proliferation results in a high amount of newly generated neuronal cells. After finishing mitosis, these granular cells start to extend their parallel fiber axons bilaterally and then start to migrate through the developing molecular layer to the internal granular layer at postnatal day 3. Additionally, during the first 3 postnatal weeks, cells in the pia matter play a role in migration and differentiation of the granular cells. Also, the Bergmann glia cells extend into the molecular layer to guide the developing granular cells to their final destination. When the granular cells reach the internal germinal layer, an extension of their dendrites into glomeruli followed by establishing contacts between excitatory afferent mossy fibers and inhibitory Golgi cell axons is generated. After that, a certain amount of the granular cell population is reduced by apoptosis within the first 3 postnatal weeks [92]. The reason for this seems to be an improvement in connectivity between the cells [93]. In addition, the Purkinje cells develop a further outgrowth and a refinement of their dendrites. While the granular cells migrate into the internal germinal layer, a connection by parallel fibers at the top of the molecular layer stacked on earlier formed parallel fibers is established. Hence, the newly formed parallel fibers stack at the top of the molecular layer, a gradient is maintained throughout the molecular layer with mature parallel fibers at the bottom and immature parallel fibers at the top. Consequently, the Purkinje cells receive two excitatory inputs from the parallel and climbing fibers. For the distal dendrites of the Purkinje cells, each spine forms a synaptic connection with an extending parallel fiber, whereas the proximal dendrites establish synaptic connections with the climbing fibers. The synaptogenesis has its peak in the second and third postnatal week [94,95].

Therefore, the Purkinje cells generate the sole output from the cerebellar cortex to the cerebellar nuclei. However, a few synapses between parallel fibers and basket cells are generated at the end of the first postnatal week. Interestingly, an important aspect of the synapse formation by parallel fibers on Purkinje cell dendrites is the presence of coated vesicles in the dendrites that appear to be endocytosing parallel fiber membrane [96,97]. The neurotransmitter released by parallel fibers is the excitatory amino acid glutamate [98].

The input from the mossy fibers to the granular layer (one of the major inputs to Ce) appears at about P5 for the first time [99]. At P12, a completed synapse formation by mossy fiber terminals on granule cell dendrites is detectable. Simultaneously, Golgi cells start to create their inhibitory synaptic connections on granule cell dendrites in the glomerulus. During the third postnatal week, the glomerulus increases its complexity and reveals an increase in the size of the mossy fiber terminal up until P18. For the inhibitory interneurons (stellate cells, basket cells and Golgi cells), they proliferate and migrate during the first or second postnatal week to their proper positions. The proliferation and differentiation of other cell types (oligodendrocytes) combined with myelination in the Ce (internal glomerular layer) starts to appear by P10 and progresses by the third postnatal week. Also, other types of cells like astrocytes seem to play an additional role in the development of the Ce [93,100]. Morphological changes for the synaptic junctions in the cerebellar glomeruli, for example, can also be determined for the matured and old mammalian central nervous system. Here, an ongoing synaptic turnover which includes formation- and consolidation changes combined with the degeneration of synaptic contact zones occurs [101].

**Transport Proteins**

The transport proteins encompass five proteins which are up-regulated and 10 down-regulated proteins towards P90. An example for the up-regulated proteins, dynactin subunit 2 (Dctn2), presents a subunit of a macromolecular complex, consisting of 10–11 subunits and capable of binding to both microtubules and cytoplasmic dynein. It is involved in diverse cellular functions, including ER-to-Golgi transport, the centripetal movement of lysosomes and endosomes, spindle formation, chromosome movement, nuclear positioning, and axonogenesis. Another up-regulated protein is serum albumin (Alb)* a soluble, monomeric protein which comprises about one-half of the blood serum protein. Alb functions primarily as a carrier protein for steroids, fatty acids, and thyroid hormones and plays a role in stabilizing extracellular fluid volume. Also, the selenium-binding protein 1 (Selenbp1)* is up-regulated at this stage. It may play a selenium-dependent role in ubiquitination/deubiquitination-mediated protein degradation. ATPase V0 subunit d1 (Atp6v0d1)) could also be detected as up-regulated at this developmental stage. This protein is responsible for acidifying a variety of intracellular compartments in eukaryotic cells, thus providing most of the energy required for transport processes in the vacuolar system. For the down-regulated proteins for example, members of the soluble NSF attachment protein family (alpha-soluble NSF attachment Protein (NAPA)*, beta-soluble NSF attachment Protein (NAPB)*) show a lower amount of expression compared to P90. These proteins play a role in the completion of membrane fusion and are required for vesicular transport between the endoplasmic reticulum and the Golgi apparatus. Also, the vesicle-fusing ATPase (Nsf)* is required for vesicle-mediated transport. It catalyzes the fusion of transport vesicles within the Golgi cisternae and is

also required for transport from the endoplasmic reticulum to the Golgi stack. It seems to function as a fusion protein required for the delivery of cargo proteins to all compartments of the Golgi stack independent of vesicle origin.

**Proteins of the Carbohydrate Metabolism**

The proteins of the carbohydrate metabolism show 10 proteins which are down-regulated, for example the protein glyceraldehyde-3-phosphate dehydrogenase (GAPDH) which catalyzes an important energy-yielding step in carbohydrate metabolism, the reversible oxidative phosphorylation of glyceraldehyde-3-phosphate as well as the regulation of mRNA stability and acting as a transferring receptor on the cell surface of macrophages. Also, members of the enolase protein family show a down-regulation (alpha-enolase (Eno1), gamma-enolase (Eno2)) which are part of a multifunctional enzyme that, as well as its role in glycolysis, plays a part in various other processes such as growth control, hypoxia tolerance and allergic responses. They may also function as an activator of plasminogen on the cell surface of several cell-types such as leukocytes and neurons. An amount of four proteins are up-regulated, for example the protein phosphoglucomutase 2-like 1 (Pgm2l1) which catalyzes the conversion of the nucleoside breakdown products ribose-1-phosphate and deoxyribose-1-phosphate to the corresponding 5-phosphopentoses. Another up-regulated protein, alcohol dehydrogenase (Akr1a1)* is responsible for the catalyzation of the NADPH-dependent reduction of a variety of aromatic and aliphatic aldehydes to their corresponding alcohols. It also catalyzes the reduction of mevaldate to mevalonic acid and of glyceraldehyde to glycerol.

**Proteins of the Energy Metabolism**

For the proteins of the energy metabolism, nine proteins are down-regulated in P7 compared to P90. For the down-regulated proteins, for example, different subunits of the ATP synthase (ATP synthase subunit alpha (Atp5a1), ATP synthase subunit d (Atp5h)) showed a differential expression. These proteins participate in the catalyzation of ATP synthesis. The ATP synthase is composed of two linked multi-subunit complexes: a soluble catalytic core and the membrane-spanning component which include the different subunits. Also, for other protein groups, a down-regulation could be analyzed. Here, different members of the mitochondrial membrane respiratory chain (NADH dehydrogenase (Complex I), NADH dehydrogenase [ubiquinone] 1 alpha subcomplex subunit 10 (Ndufa10), NADH-ubiquinone oxidoreductase 75 kDa subunit (Ndufs1)*, NADH dehydrogenase [ubiquinone] iron-sulfur protein 2 (Ndufs2), NADH dehydrogenase (Ubiquinone) Fe-S protein 3 (Ndufs3)) show a differential expression. These proteins are subunits of the mitochondrial membrane respiratory chain NADH dehydrogenase (Complex I) that is believed to belong to the minimal assembly required for catalysis. This complex functions in the transfer of electrons from NADH to the respiratory chain. Also, different types of the creatine kinase (creatine kinase B-type (Ckb)*, creatine kinase U-type (Ckmt1)*) could be identified as down-regulated. These are involved in energy homeostasis and reversibly catalyze the transfer of phosphate between ATP and various phosphogens such as creatine phosphate.

**Chaperones**

The category of chaperones shows an amount of five up-regulated and four down-regulated proteins. For the up-regulated proteins, these contain subunits of the chaperonin containing TCP1 complex (CCT) which acts as a molecular chaperone and assists the folding of proteins upon ATP hydrolysis. Another up-regulated protein, the 60 kDa heat shock protein (Hspd1)* functions as a mitochondrial protein importer and macromolecular assembly. It also may facilitate the correct folding of imported proteins and promote the refolding and proper assembly of unfolded polypeptides generated under stress conditions in the mitochondrial matrix. The down-regulated proteins contain different members of the heat shock protein family (heat shock protein HSP 90-beta (Hsp90ab1), heat shock 70 kDa protein 4 (Hspa4)*, heat shock 70 kDa protein 5 (Hspa5)). While Hsp90ab1 belongs to the heat shock protein 90 family which is involved in signal transduction, protein folding and degradation, Hspa4 and Hspa5 are members of the heat shock protein 70 family which are also involved in the folding and assembly of proteins in the ER. As these proteins interact with many ER proteins, they may also play a key role in monitoring protein transport through the cell.

**Proteins of the Signal Transduction**

The proteins of the signal transduction encompass six proteins which are expressed differentially. An amount of three proteins of this category are up-regulated. For example, guanine nucleotide-binding protein G(q) subunit alpha (Gnaq)* which acts as a modulator in various transmembrane signaling systems. Other up-regulated proteins are members of the 14-3-3 protein family (14-3-3 protein epsilon (Ywhae), 14-3-3 protein zeta/delta (Ywhaz)*) which mediates signal transduction by binding to phosphoserine-containing proteins. They participate in the regulation of a large spectrum of both general and specialized signaling pathways. For the down-regulated proteins, also here, members of 14-3-3 protein family show a differential expression ((14-3-3 protein eta (Ywhah)*, 14-3-3 protein theta (Ywhaq)*). In addition, inositol monophosphatase 1 (Impa1) also displays a down-regulation, it functionally acts as an important modulator of intracellular signal transduction via the production of the second messenger myoinositol 1,4,5-trisphosphate and diacylglycerol.

**Proteins of the Amino Acid Metabolism**

The proteins of the amino acid metabolism show four proteins which are down-regulated, for example glutamine synthetase (Glul)*. It catalyzes the synthesis of glutamine from glutamate and ammonia. Glutamine is a main source of energy and is involved in cell proliferation, inhibition of apoptosis, and cell signaling. Another down-regulated protein, aspartate aminotransferase (Got1)* plays an important role in the amino acid metabolism and the urea of tricarboxylic acid cycles. Additionally, it is an important regulator of levels of glutamate and acts as a scavenger of glutamate in brain neuroprotection. An up-regulation was only detectable for one member of this protein category (GMP synthase (Gmps)). Gmps is involved in the de novo synthesis of purine nucleotides for the synthesis of either guanine or adenine nucleotides. One protein (branched-chain-amino-acid aminotransferase (Bcat1)*) is absent at P90 compared to P7. This protein is responsible for the reversible transamination of branched-chain alpha-keto acids to branched-chain L-amino acids and therefore is essential for cell growth.

**Degratory Proteins**

The proteins of degradation reveal six proteins which are up-regulated. The up-regulated faction contains different members (Proteasome subunit beta type-3 (Psmb3), proteasome subunit alpha type-6 (Psma6), proteasome subunit beta type-7 (Psmb7), proteasome 26S subunit 7 (Psmd7)*, proteasome 26S subunit 14 (Psmd14)) of the proteasome. The Members of this multicatalytic proteinase complex cleave peptides in an ATP/ubiquitin-dependent process in a non-lysosomal pathway.

**Proteins with Antioxidant Capacity**

Proteins with antioxidant capacity exhibit a down-regulation of protein expression towards P90 (4 proteins). These contain glutathione *S*-transferase Mu 5 (Gstm5) and glutathione *S*-transferase omega-1 (Gsto1), belonging to the mu class. The mu class of enzymes functions in the detoxification of electrophilic compounds, including carcinogens, environmental toxins and products of oxidative stress. Others are the proteins thioredoxin-dependent peroxide reductase (Prdx3) and peroxiredoxin-6 (Prdx6). Both are members of the thiol-specific antioxidant protein family and are involved in the redox regulation of the cell by reducing $H_2O_2$ and short chain organic, fatty acid, and phospholipid hydroperoxides. They may play a role in the regulation of phospholipid turnover as well as in protection against oxidative injury. *N*(G),*N*(G)-dimethylarginine dimethylaminohydrolase 1 (Ddah1) is the only protein from this category which is up-regulated in P7 compared to P90. It belongs to the dimethylarginine dimethylaminohydrolase (DDAH) gene family and plays a role in nitric oxide generation by regulating cellular concentrations of methylarginines, which in turn inhibit nitric oxide synthase activity.

**Proteins of the Fat Metabolism**

The proteins which are involved in fat metabolism show a down-regulation of one protein and an up-regulation of two proteins. Phytanoyl-CoA hydroxylase-interacting protein (Phyhip), the up-regulated protein, interacts with phytanoyl-CoA 2-Hydroxylase (Phyh) which is involved in the alpha-oxidation of 3-methyl branched fatty acids. Its interaction with Phyh suggests a role in the development of the central nervous system.

**Regulatory Proteins**

The proteins which are involved in regulatory processes display an amount of three proteins which are up-regulated and four down-regulated proteins. In addition, one protein is absent at P90 compared to P637. For the up-regulated proteins, aspartoacylase (Aspa)* catalyzes the conversion of *N*-acetyl_L-aspartic acid to aspartate and acetate. In the brain, the hydrolysis function by aspartoacylase is thought to help maintain white matter. Another example is constitutive photomorphogenic homolog subunit 5 (Cops5). It is one of the subunits of COP9 signalosome, a protein complex that functions as an important regulator in multiple signaling pathways and is reported to be involved in the degradation of cyclin-dependent kinase inhibitors. The down-regulated proteins include clathrin light chain B (Cltb), a large, soluble protein composed of heavy and light chains. It functions as the main structural component of, for example, vesicles which entrap specific macromolecules during receptor-mediated

endocytosis. Also, SET nuclear oncogene (Set)* which is a multitasking protein, involved in apoptosis, transcription, nucleosome assembly and histone chaperoning is down-regulated at this stage. Snx4 protein (Snx4)*, a member of the sorting nexin family, is absent at P90 compared to P637. Members of this family are involved in intracellular trafficking.

**Structural Proteins**

The structural proteins display four proteins which are up-regulated in comparison to P90. Besides the dihydropyrimidinase-related protein 2 (Dpysl2)* which plays a role in axon guidance, neuronal growth cone collapse and cell migration, also members of the tubulin protein family (tubulin alpha-1B chain (Tuba1b)*, tubulin beta-3 chain (Tubb3)*) show a differential expression towards P90. These proteins are one of two core protein families (alpha and beta tubulins) that heterodimerize and assemble to form microtubules. These proteins are primarily expressed in neurons and may be involved in neurogenesis and axon guidance and maintenance. An amount of three proteins show a down-regulation towards P90. Dynamin-1 (Dnm1)*, a member of the dynamin subfamily of GTP-binding proteins, is involved in clathrin-mediated endocytosis and other vesicular trafficking processes while actin and other cytoskeletal proteins act as binding partners for the protein. Also, two members of the septin family (septin-11 (Sept11), septin-5 (Sept5)*) are expressed differentially. Both of them are responsible for filament-forming cytoskeletal GTPases that are involved in a variety of cellular functions including cytokinesis and vesicle trafficking.

**Transport Proteins**

The transport proteins present six proteins which are down-regulated towards P90. AP-2 complex subunit beta (Ap2b1)* is a component of the adaptor protein complex 2 (AP-2). It functions in protein transport via transport vesicles in different membrane traffic pathways, cargo selection and vesicle formation. Furthermore, dynein Intermediate chain 2 (Dync1i2), an accessory component of the cytoplasmic dynein 1 complex and involved in linking dynein to cargos and to adapter proteins that regulate dynein function also displays a down-regulation. Also, the vesicle-fusing ATPase (Nsf)* is required for vesicle-mediated transport and shows a down regulation as well. It catalyzes the fusion of transport vesicles within the Golgi cisternae and is also required for transport from the endoplasmic reticulum to the Golgi stack.

**Proteins Involved in Signal Transduction**

The proteins which are involved in the pathways of the signal transduction show two proteins which are up-regulated. Guanine nucleotide-binding protein (G(I)/G(S)/G(T) subunit beta-1 (Gnb1)*, guanine nucleotide-binding protein G(I)/G(S)/G(T) subunit beta-2 (Gnb2)*). Both of these subunits of G proteins are involved as modulators or transducers in various transmembrane signaling systems. The beta chains are required for the GTPase activity, for replacement of GDP by GTP, and for G protein-effector interaction. Furthermore, an amount of three down-regulated proteins could be analyzed (14-3-3 protein eta (Ywhah)*, 14-3-3 protein theta (Ywhaq)*, 14-3-3 protein zeta/delta (Ywhaz)*). These are members of the 14-3-3 protein family are a group of highly conserved proteins that are involved in many vital

cellular processes such as metabolism, protein trafficking, signal transduction, apoptosis and cell cycle regulation.

### Proteins of the Energy Metabolism

The proteins involved in energy metabolism show a down-regulation of three proteins. These include creatine kinase B-type (Ckb)* and creatine kinase U-type (Ckmt1)*. As described above, both are involved in energy homeostasis by reversible catalyzation of phosphate between ATP and various phosphogens. Additionally, NADH-ubiquinone oxidoreductase 75 kDa subunit (Ndufs1)*, a core subunit of the mitochondrial membrane respiratory chain NADH dehydrogenase (Complex I) which functions in the transfer of electrons from NADH to the respiratory chain, also displays a down-regulation. On protein could be identified as up-regulated towards P90 (ATP synthase subunit alpha (Atp5a1). This protein is a subunit of the mitochondrial ATP synthase. Therefore it is participating in ATP synthesis, using an electrochemical gradient of protons across the inner membrane during oxidative phosphorylation.

### Proteins of the Amino Acid Metabolism

In this category of proteins involved in the amino acid metabolism, only one protein could be analyzed which shows a differential expression. This protein, cytosolic non-specific dipeptidase (CNDP2) hydrolyzes a variety of dipeptides including L-carnosine.

### Proteins of the Fat Metabolism

The proteins involved in fat metabolism only show one differentially expressed protein, acetyl-CoA acetyltransferase (Acat2). This up-regulated protein is involved in lipid metabolism. It catalyzes the acetylation of acetyl-Coenzym A which is important for the assembly of keto bodies, but is also involved in the biosynthesis of steroids and for the disassembly of some amino acids.

### Degratory Proteins

The last category, the degratory proteins present one protein (ubiquitin carboxyl-terminal hydrolase (Usp5)*) which is up-regulated in comparison to P90. The ubiquitin dependent proteolysis is a complex pathway of protein metabolism implicated in cellular functions as maintenance of chromatin structure, receptor function and degradation of abnormal proteins. A late step of the process involves disassembly of the polyubiquitin chains on degraded proteins into ubiquitin monomers

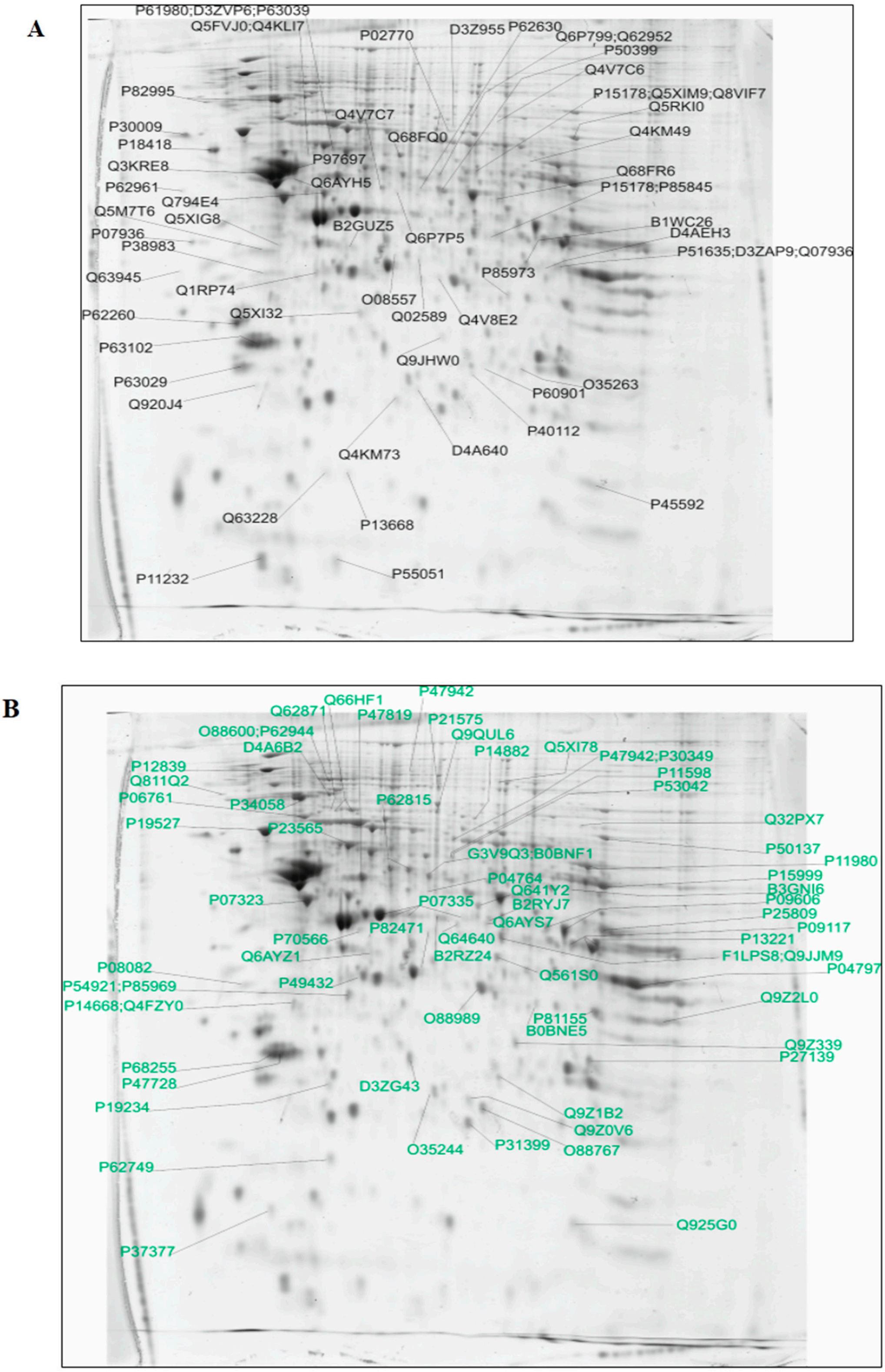


**Figure S2.** *Cont.*

C

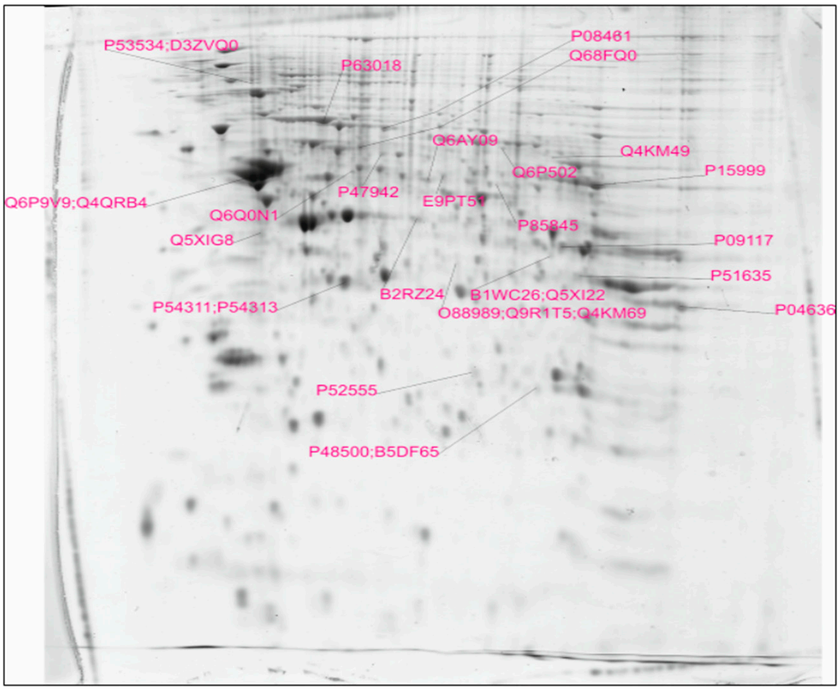


D

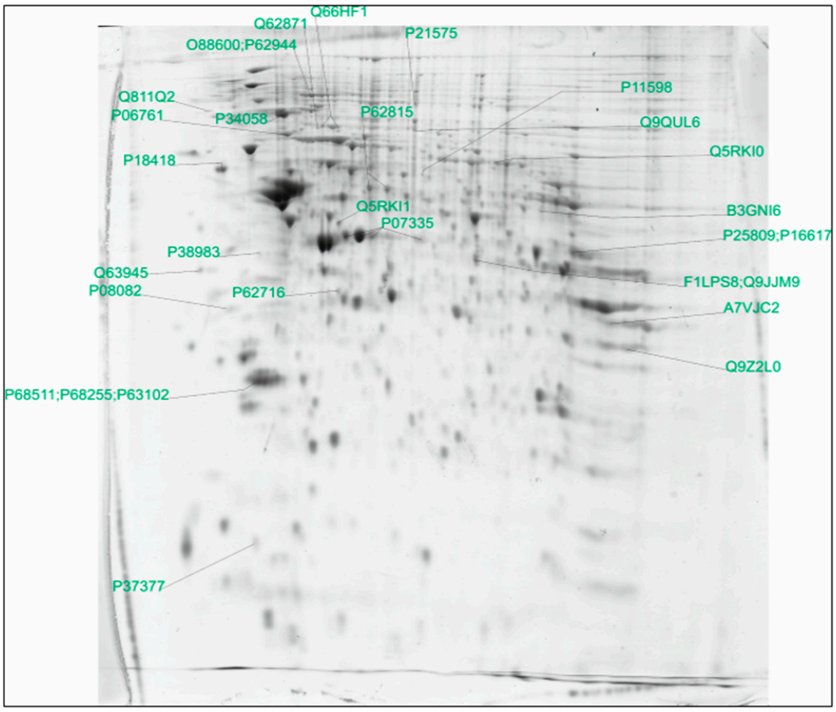


**Figure S2.** Annotation of accession numbers from Swiss Prot in the reference gel (P90, reference gel: Cerebellum-P90-LK-4-6). Protein mixes and multiple protein presentations in different spots can be derived from branching lines. (**A**) differential expression of P7 in comparison to P90 (black: up-regulated proteins); (**B**) differential expression of P90 in comparison to P7 (green: up-regulated proteins); (**C**) differential expression of P637 in comparison to P90 (pink: up-regulated proteins); (**D**) differential expression of P90 in comparison to P637 (green: up-regulated proteins).
